# *Larrea divaricata*: anti-inflammatory and antioxidant effects of on macrophages and low density lipoproteins

**DOI:** 10.1186/s12906-022-03547-8

**Published:** 2022-03-23

**Authors:** Ignacio Peralta, Carla Marrassini, Malen Saint Martin, Laura Cogoi, María Rosario Alonso, Alejandro Gugliucci, Claudia Anesini

**Affiliations:** 1 Universidad de Buenos Aires, Consejo Nacional de Investigaciones Científicas y Técnicas (CONICET), Instituto de Química y Metabolismo del Fármaco (IQUIMEFA), Buenos Aires, Argentina; 2 Cátedra de Farmacognosia, Facultad de farmacia y Bioquímica, Universidad de Buenos Aires, Buenos Aires, Argentina; 3 Disease Laboratory, Touro University of California, University of Touro, New York, CA USA

**Keywords:** *Larrea divaricata*, NDGA, Glucose, HDL, LDL

## Abstract

**Background:**

The oxidized low density lipoprotein (ox-LDL) contributes to inflammation and oxidative stress through the activation of macrophages under hyperglycemia contributing to the development of diabetes mellitus and to atherosclerosis. Plants are a source of effective and innocuous antioxidants. *Larrea divaricata* Cav. (Zygophyllaceae) is used in Argentina folk medicine for its anti-inflammatory properties.

**Methods:**

The aim of this work was to study the antioxidant and anti-inflammatory effects of the aqueous extract (AE) of *L. divaricata* on macrophages under glucose stimulation and on human LDL and HDL particles under free radical generators.

**Results:**

AE reduced the lipid peroxidation (17%), nitric oxide (NO) (47-50%), tumor necrosis factor-α (TNF-α) (32%) and free radicals (50%) induced by glucose on macrophages. Also prevented HDL nitration (28%), thus preserving its function and structure and inhibited LDL oxidation. The effect on the nitrosative stress was mainly driven by nordihydroguaiaretic acid (NDGA).

**Conclusions:**

These results suggest a potential usefulness of AE as an adjuvant phytotherapy in patients with diabetes mellitus and atherosclerosis.

**Supplementary Information:**

The online version contains supplementary material available at 10.1186/s12906-022-03547-8.

## Background

Hyperglycemia has been identified as the main contributing factor to diabetes (DM)-associated atherosclerosis. Atherosclerosis is initiated by the oxidation and glycosylation of the apolipoprotein B (Apo-B) contained in low density lipoprotein (LDL) particles, which transport cholesterol and triglycerides to tissues. Oxidized LDL is phagocytosed by macrophages to generate foamy cells containing cholesterol and triglycerides, initiating an inflammatory and oxidative stress response [1]. This cascade of events gives rise to an atheromatous plaque that blocks the blood flow. The process described above is counteracted by the action of HDL particles, Apo-A1 and Apo-E, which inhibit inflammation and oxidative stress. However, if HDL particles cannot exert their protective functions, the atherogenic process perpetuates itself leading to cardiovascular ischemic events [2].

Since the antioxidant systems may sometimes be insufficient, the administration of exogenous innocuous antioxidant substances may be required to avoid the diabetic complications. The current trend is to search for antioxidant substances with the capacity to modulate blood lipid levels. These substances may be found in plant extracts, which have been consumed for generations and generally are being well tolerated y people possess which present fewer adverse effects than synthetic compounds. These substances may be found in plant extracts, which have been consumed for generations and, when used in a correct dose, were generally better tolerated than synthetic compounds.

*Larrea divaricata* Cav. (Zygophyllaceae) is a bush that grows in South America and is widely distributed in Argentina. This plant is used in folk medicine for its anti-inflammatory properties and is also known to have antitumoral, immunomodulatory and antimicrobial properties [3–6]. The presence of the antioxidant compound NDGA has already been reported in this plant [7].The aim of this work was to study the effects of AE and its majority compound, NDGA, on the lipid peroxidation and the production of nitric oxide (NO), free radicals, reduced glutathione (GSH), and TNF-α in macrophages cultured under glucose stimulation, simulating as diabetic status. The effects of AE and NDGA were also studied on the antioxidant activity and on the structure of HDL-paraxonase (PON-1) treated with 3-morpholinosydnonimine (SIN-1) and 2,2’-azobis (2-amidinopropane) dihydrochlorohydrate (AAPH) and on Cu^2+^-induced LDL oxidation.

## Methods

### Plant material

Leaves of *Larrea divaricata* Cav. were collected in the province of Córdoba, Argentina (Rio Dolores district, Barrio Aguas Azules, Sector B, Capilla del Monte, Punilla Department, province of Córdoba, Argentina, land register data: Dep. 23- Ped. 01- Pueblo 06- Circ. 05- Secc. 02- Manzana 054- Parcela 003). No state permissions were necessary to collect the sample. The plant material was identified by morphological, anatomical and histochemical criteria by Dr. Hernán Gerónimo Bach from the Museum of Pharmacobotany, School of Pharmacy and Biochemistry, University of Buenos Aires. One voucher specimen (BAFC no. 38) was deposited at the Museum of Pharmacobotany. The aqueous extract (AE) was prepared from air-dried leaves. Briefly, 750 mg of leaves were infused for 20 min with 10 ml of sterilized boiling distilled water and the supernatant was lyophilized [8]. The plant material was processed following the World Health Organization (WHO) guidelines on Good Agricultural and Collection Practices (GACP) for medicinal plants (WHO, 2003) [9].

### NDGA quantification by HPLC and characterization of the extract

The high performance liquid chromatography (HPLC) analysis was performed in a Varian Pro Star instrument equipped with a Rheodyne injection valve (20 µl) and a photodiode array detector set at 280 nm. A reversed-phase Phenomenex-C18 (2) Luna column (250 mm x 4.6 mm and 5 µ pd) was used. Samples were eluted with a gradient of A: water:acetic acid (98:2) and B: methanol:acetic acid (98:2) from 15% B to 40% B in 30 min; 40% B to 75% B in 10 min; 75% B to 85% B in 5 min and 100% B in 5 min. Solution B (100%) was run for 10 min and back to initial conditions. The flow rate was 1.2 ml/min and the separation was done at room temperature (18-25ºC). The optical density of eluates was registered in a Varian Star 5.5 detector (USA). Lyophilized aqueous extracts (10 mg/ml) and the pure standard were dissolved in methanol:water (70:30). The water employed to prepare the working solution was of ultrapure quality (Milli-Q). Methanol (J.T. Baker) and acetic acid (Merck, Argentina) were HPLC grade. NDGA (Sigma, USA, lot 19 C-0504, >97.0% purity), 4-hydroxybenzoic acid (4-HBA, Sigma, USA, ≥99% purity) and rutin (Sigma, USA, ≥94% purity) standards were employed [8].

### Total polyphenol determination

The total polyphenols content was determined by spectrophotometry by the Folin-Ciocalteu’s method using gallic acid as standard. The lyophilized extract was weighed and dissolved in distilled water. Briefly, 1.0 ml of the extract was transferred to separate tubes containing 7.0 ml of distilled water, 0.5 ml of Folin–Ciocalteu’s reagent, and 1.5 ml of a 20% sodium carbonate anhydrous solution. Tubes were allowed to stand at room temperature for 60 min and the absorbance at 765 nm was measured in a UV-vis spectrophotometer. The concentration of polyphenols in the samples was derived from a standard curve of gallic acid ranging from 10 to 50 µg/ml (Pearson’s correlation coefficient: *r*^2^ = 0.9996) [10].

### Macrophages culture

 The RAW264.7 murine macrophage cell line was purchased from American Type Culture Collection (ATCC) and kindly provided by Dr Renzo Martino from the Immunology Laboratory, Immunology Unit, Faculty of Pharmacy and Biochemistry, University of Buenos Aires.

Cells (5 × 10^5^ cells/ml) were cultured in Dulbecco’s modified Eagle’s medium supplemented with 10% fetal bovine serum containing antibiotics (100 IU/ml penicillin and 100 µg/ml streptomycin) and kept in a humidified incubator at 5% CO_2_ and 37 °C [11].

### Antioxidant activity tests in macrophage cultures

The diagnostic criterion for diabetes is a blood glucose concentration of >100 mg/dl. Therefore, in the present study, 5.5 mM glucose (100 mg/dl) was used as control, and 11mM and 55 mM glucose were used as high-glucose levels to emulate diabetic status in macrophages.

### Determination of cell proliferation and viability

To evaluate the proliferation of RAW macrophages, the MTT (3- (4,5-dimethylthiazol-2-yl) -2,5-diphenyltetrazolium bromide) technique was used to measure cell proliferation. Cells (2 × 10^5^ cells/ml) were incubated in 96-well plates in the presence of glucose with or without AE or NDGA. AE was prepared in distilled water and NDGA (Sigma, USA, lot 19 C-0504, >97.0% purity) was dissolved in ethanol:water (stock solution) and further diluted in culture media. The final concentration of ethanol in the wells did not exceed 0.5%. The extract, the compounds or glucose were added to culture at the same time and incubated during 24 h. Cells were then washed and incubated with 5 mg/ml MTT (Sigma) for 4 h. The amount of formazan formed, which is proportional to the number of cells in the well, was extracted with acidic isopropanol, and the absorbance was measured in a microplate reader at 540 nm. Results were expressed as absorbance [12]. Viability was determined by the trypan blue dye exclusion method. Briefly, the cell suspension was mixed with 0.4% trypan blue in a 1: 9 ratio and the mixture was transferred to a Neubauer hemocytometer to determine the number of viable and non-viable cells. Viability was expressed as percentage of viable cells, calculated according to the following formula:% viable cells=number of viable cells x 100/total number cells [11].

### Determination of NO

To assess the production of NO, macrophages were incubated for 24 h with glucose (basal: 5.5 mM, 11 mM, 55 mM and 110 mM) with or without different concentrations of AE or NDGA. Cells were then centrifuged at 900 *xg* for 10 min. After centrifugation, the supernatant was removed to determine the levels of total nitrites (TN) as indicator of NO produced and the pellet was used to determine the cell count. The total accumulated nitrites in the culture medium, was measured by the Griess reaction [13]. Briefly, 100 µl of each supernatant were mixed with 50 µl of a 1% sulfanilamide solution in 5% phosphoric acid and 50 µl of a 0.1% naphthylenediaminedihydrochloride solution. The mixture was then incubated at room temperature for 10 min, and the absorbance was read at 550 nm. As a standard curve, serial dilutions (0-100 µM) of a NaNO_2_ solution were used. Results were expressed as TN in nM/10^6^ cells.

### Determination of intracellular reactive oxygen species (ROS)

The production of total ROS was determined by the addition of the 2´,7´-dichlorodihydrofluorescein diacetate (DCFH-DA), which is oxidized by the ROS present in the sample to 2´,7´-dichlorofluorescein (DCF), a fluorescent compound whose fluorescence intensity was assessed by flow cytometry. Cells were incubated with increasing concentrations of glucose (5.5 mM, 11 mM, and 55 mM) in either the presence or absence of AE (0 µg/ml, 0.1 µg/ml and 1 µg/ml) or NDGA (0 µg/ml, 0.0003 µg/ml, 0.003 µg/ml). More than 10,000 events per group were acquired on a FACS Calibur flow cytometer, and the mean population cell fluorescence intensity (MFI) was obtained from histogram charts. Results were expressed as DCF-DA (MFI).

### Determination of TBARS

The concentration of TBARS (Thiobarbituric acid reactive substances), which represent lipoperoxides formed in macrophage cultures, was determined in the presence of different concentrations of glucose with or without AE or NDGA. Sample supernatants (20 µl) were mixed with 600 µl of the freshly prepared reagent containing 0.046 mol / L thiobarbituric acid. After incubating for 60 min at 95 °C, samples were cooled over ice, centrifuged at 1000 *xg* for 15 min at 4 °C and the supernatant absorbance was read at 532 nm. The absorbance is proportional to the amount of TBARS present in the sample. 1,1,3,3, Tetramethoxypropanebis(dimethyl acetal) malondialdehyde was used as standard [14, 15].

### Determination of tumor necrosis factor α (TNF-α) levels

After 24 h of incubation, the production of TNF-α was determined in macrophages treated with 5.5 mM (basal) and 55 mM glucose in either the absence or in the presence of different concentrations of AE or NDGA. TNF-α levels were measured in the cell culture supernatant using a commercial kit (mice TNF-α ELISA KIT, Chemicon International, Inc., CA, USA).

### Determination of reduced glutathione (GSH) levels

GSH levels were determined according to Moron et al. (1979) [16] in cultures treated with different glucose concentrations in either the presence or the absence of different concentrations of AE and NDGA. Briefly, immediately after separation, the supernatants were precipitated with 0.1 ml of 25% TCA. Samples were then centrifuged and the precipitate was removed. Free sulfhydryl groups were determined in a total supernatant volume of 200 µl. Briefly, 134 µl of a 0.6 mM DTNB (5.5´-dithio-bis-(2-nitrobenzoic acid, Sigma) solution and 56 µl of 0.2 mM sodium phosphate buffer (pH 8.0) were added to 10 µl of each supernatant and the absorbance was read at 405 nm on a Systronics UV-VIS spectrophotometer. Glutathione (Sigma) was used as standard. Results were expressed as µM GSH / 10^6^ viable cells.

### Determination of cytotoxicity

The cytotoxic effect on macrophages was determined upon incubation with a peroxynitrite generator (3-morpholinosydnonimine, SIN-1; Sigma, USA) and a ROS generator (2,2’-azobis (2-amidinopropane) dihydrochloride, AAPH; Sigma, USA) in either the absence (basal) or the presence of different concentrations of either AE or NDGA(Sigma, USA, lot 19 C-0504, >97.0% purity). AAPH and SIN-1 were dissolved in distilled water. The Thermo Scientific™ Pierce™ LDH Cytotoxicity Assay Kit (cat. no. 88,954) (Rochford, USA)was used to determine the lactate dehydrogenase (LDH) activity in culture media using an enzymatic reaction that produces a red formazan product that is measured at 490 nm. The presence of cytosolic LDH in the culture supernatant is due to cell membrane damage. The activity measured under basal conditions was taken as 100% viability. The percentage of viability and cell cytotoxicity were calculated as follows: % viability= [U/ml treated x 100]/U/ml basal. %cytotoxicity: 100-%viability [17].

### Effects on plasma lipoproteins

#### Preparation of plasma and lipoproteins and isolation of LDL and HDL particles

Blood was obtained from eight healthy and normolipemic volunteers by venipuncture and collected in vacuum system tubes with ethylenediaminetetraacetic acid (EDTA, 5mmol/L). Blood samples were centrifuged at 800 *xg*, at 4 °C for 15 min and the plasma was used to isolate HDL and LDL particles as described below. Informed consent was obtained from all volunteers and the research was carried out in accordance with the principles of the Declaration of Helsinki. These tests were carried out at the Glycation, Oxidation and Disease Laboratory, Touro University of California, University of Touro, California, USA.

**Fig. 1:**
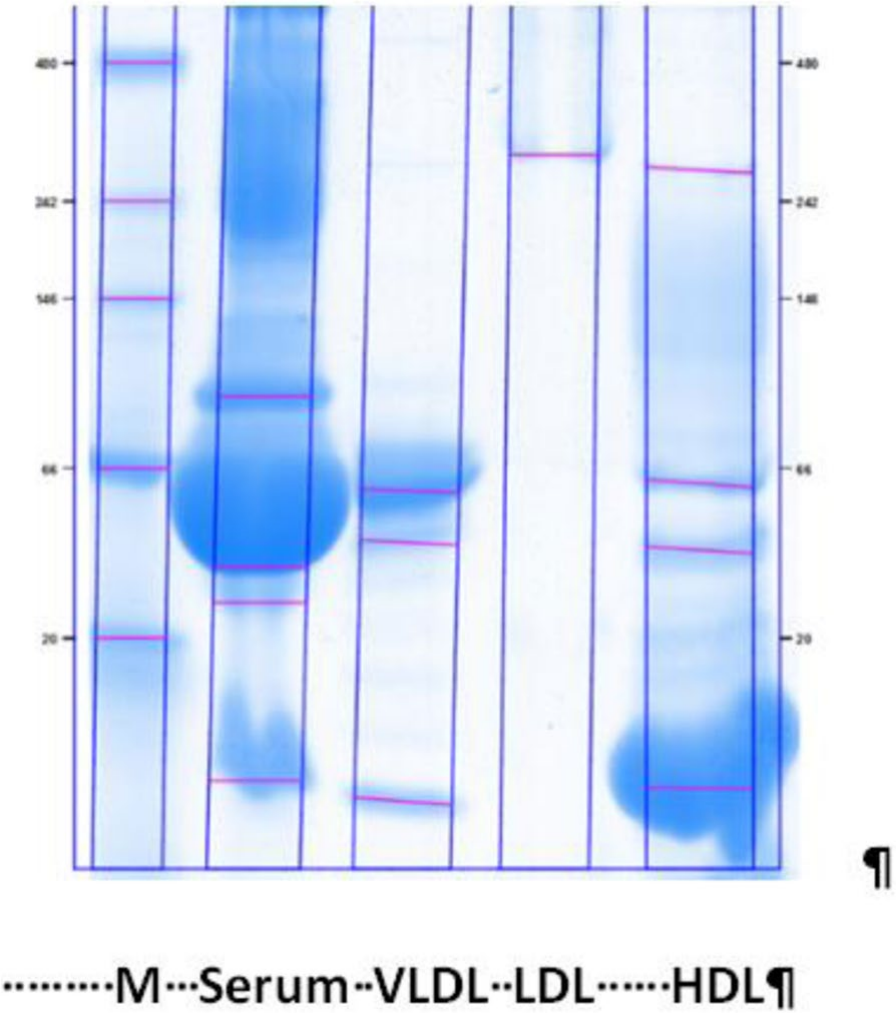
Isolation of human HDL and LDL particles by differential ultracentrifugation. M: molecular weight marker

LDL (δ = 1,019-1,063 g/ml) and HDL (δ = 1,063-1,210 g / ml) particles were obtained from plasma by sequential float ultracentrifugation at 15 °C [18]. After extensive dialysis, both HDL and LDL were maintained in a 10 mmol/L sodium phosphate buffer, pH 7.4 containing 150 mmol/L NaCl, 0.1 mmol/L EDTA at 4 °C and used within one week. Before oxidation experiments, lipoproteins were dialyzed overnight against the same buffer without EDTA. The isolated particles were then analyzed by electrophoresis and gels were stained with Coomassie blue to determine the purity of each fraction (Fig. 1) [19].

### Effect of oxidative and nitrosative stress on PON-1 activity

The aryl esterase activity of HDL-associated paraoxonase-1 (PON-1) was determined in either the presence or absence of 2,2’-azobis (2-amidinopropane) dihydrochlorohydrate (AAPH, 5mM, Sigma) or 3-morpholinosydnonimine (SIN-1, 10 µM, Sigma, USA). For each free radical generator, different concentrations of AE and NDGA (Sigma, USA, lot 19 C-0504, >97.0% purity)were tested. The aryl esterase activity was determined on a SpectraMax® microplate spectrophotometer. The rate of phenol formation was monitored every 15 Sect. (270 nm, room temperature) after the addition of 20 µl (1:80 dilution) of plasma to 200 µl of a 3.26 mmol/Lphenyl acetate solution (9 mmol/LTris-HCl, pH 8.0, 0.9 mmol/L CaCl_2_). Results were expressed in U/L using the following equation:U/L = ΔA / minute x F. WhereΔA = ΔAbsorbance; F = (TV / SV) / 0.00131; TV = Total volume in µl; SV = Sample volume in µl; 0.00131 = micromolar extinction coefficient [20].

### Westernblot analysis of Apo-A1 in the presence of SIN-1

A sodium dodecyl sulfate polyacrylamide gel electrophoresis (SDS-PAGE) was performed under denaturing conditions according to Laemmli. An HDL sample was loaded in either the absence (basal) or the presence of SIN-1 (20mM), with or without AE (0.1 and 1 µg/mL) or NDGA (0.0003 and 0.003 µg/mL). Samples were then transferred to a PVDF membrane using the Transblot SD Semidry Cell (Bio-Rad) at 15 V for 35 min. For the detection of Apo-A1, HRP-conjugated antibodies (anti-ApoA-1 (HRP) Abcam ab20784, 1:1,000 in 10% FCS) were used. Blots were then incubated with the ECL PLUS reagent (Amersham), developed and scanned in a chemiluminescent scanner (LICOR C-DIGit, LICOR Biosciences) and processed and quantified with the Image Studio software. Three independent experiments were done [20].

### Oxidation of LDL in presence of CuSO_4_and determination of TBARS

To oxidize LDL, 1 ml of the preparation adjusted to 100 mg protein/mL was incubated with 10 mmol/L sodium phosphate buffer pH 7.4 containing 150 mmol / L NaCl, in the presence of fresh 80 mMCuSO_4_. The oxidation of LDL was assessed through the determination of TBARS as described above. The oxidation protocol was carried out in either the presence or absence of different AE concentrations (0.1 µg/ml, 1 µg/ml, and 10 µg/ml) and the relative concentrations of NDGA [14].

[[The oxidized LDL preparations (500 µl) were mixed with 1 ml of the freshly prepared reagent containing 0.046 mol / L of thiobarbituric acid, 0.92 mol / L of trichloroacetic acid and 0.25 mol / L of HCl. After incubating for 30 min at 100 ° C, samples were cooled on ice, centrifuged at 1000*xg* for 15 min at 4 °C and the absorbance of the supernatant was read at 532 nm. The sample absorbance is proportional to the formation of TBARS. 1,1,3,3, Tetramethoxypropanebis(dimethyl acetal)malondialdehyde was used as standard [14, 15].

### Statistical analysis

The statistical analysis was performed with three or more independent experiments performed in triplicate. In all cases, the mean and the standard error of the mean (SEM) were determined. The significance between the means was analyzed by one way ANOVA and the Dunnett´s test for comparison of treated samples with the control. In all cases, the differences were considered significant when *p* ≤0.05. Cells were used randomly and the parameters were double-blind evaluated.

## Results

### Characterization of the extract, quantification of NDGA and total polyphenols

By HPLC-UV, NDGA presented a retention time of 47 min, and its concentration in the extract was found to be 0.30 ± 0.01 g%. Other compounds such as the phenolic acid 4-hydroxybenzoic acid (4-HBA) (retention time: 12 min), together with flavonoids such as rutin (retention time: 31.6 min) were identified but in lower amounts (4-HBA: 0.0343 ±0.003 g%, rutin:0.1 ± 0.01 g%, chromatogram not shown).

The content of total polyphenols in the extract presented was 10.68 ± 0.43 g%. All experiments were compared with NDGA, which was assayed at the concentration at which it was found in the extract.

### Anti-inflammatory and antioxidant activities of AE and NDGA on macrophages

#### Effects of AE and NDGA on the proliferation of macrophages under glucose stimulation

**Fig. 2:**
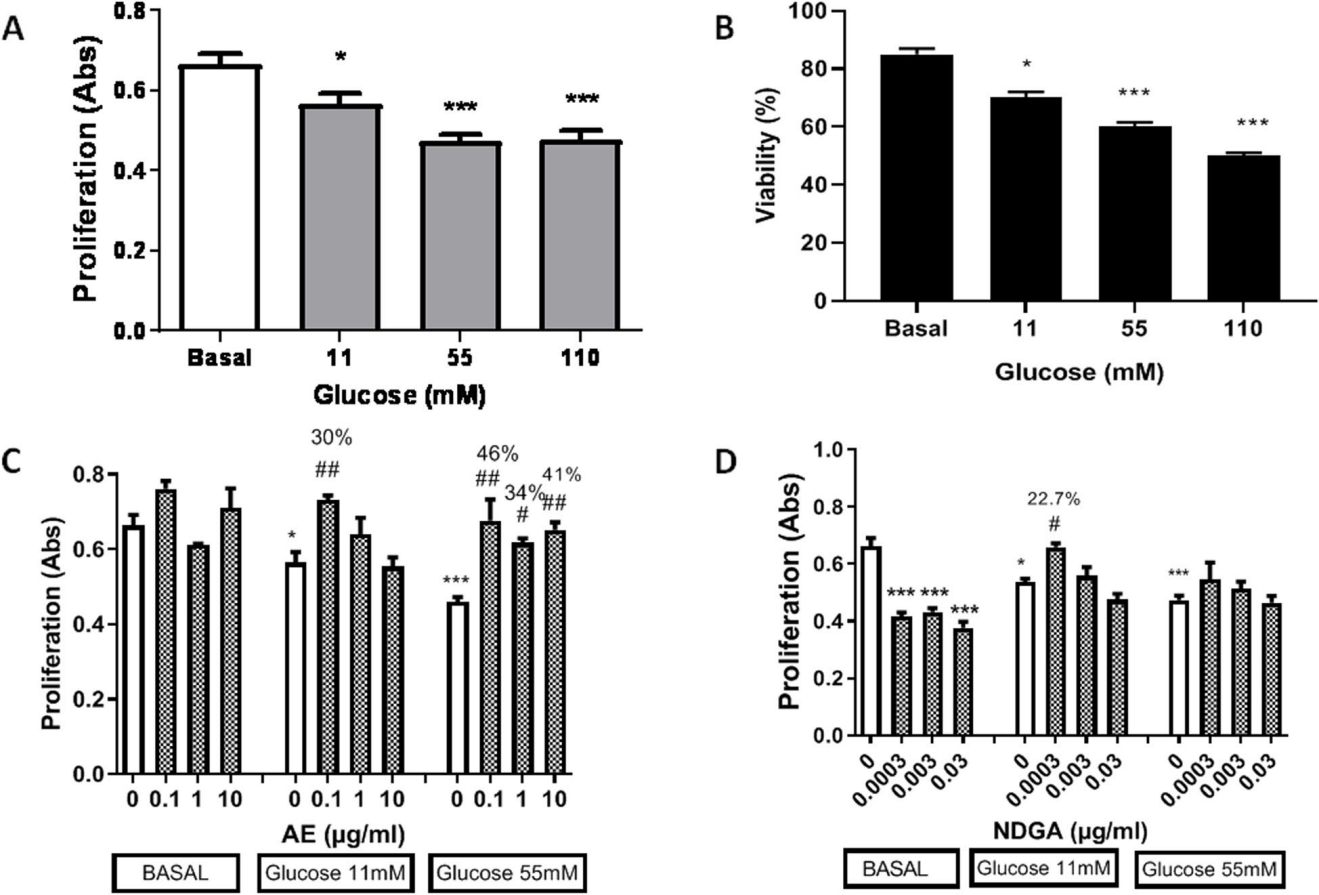
Effect of AE and NDGA on glucose-treated macrophage proliferation and viability. **A**,** B** Effect of glucose on cell proliferation and viability. **C** Effect of AE on the proliferation of cells treated with glucose. **D** Effect of NDGA on the proliferation of cells treated with glucose. Cells were incubated for 24 h in the presence of the different concentrations of glucose: 5.5 mM (basal), 11 mM, 55 mM and 110 mM with or without AE or NDGA. The percentage of reversal is indicated over the bars. Results are expressed as the Mean ± SEM of three or more experiments performed in triplicate. * *p* <0.05, ** <0.01, *** <0.001: significantly different with respect to basal conditions (5.5 mM glucose) and #p < 0.05,## *p* < 0.01, ###<0.001: significantly different with respect to each glucose concentration (One way ANOVA followed by Dunnett’s test)

The effects of AE and NDGA were studied on macrophages incubated with different concentrations of glucose. Macrophages were first incubated under different concentrations of glucose(5.5mM, basal condition; 11mM, 55 mM and 110 mM), to select the concentration to be used in subsequent experiments. Proliferation and viability were evaluated. Glucose significantly decreased cell proliferation and viability at 11mM, 55mM and 110 mM (around15-28%)in comparison with glucose 5.5 mM (basal condition, Fig. 2A and B).No significant differences were observed in the proliferation levels between 55 mMand110 mM glucose.

The effects of AE and NDGA were then evaluated alone and in the presence of11mM and 55 mM glucose. AE at 0.1 µg/ml, 1 µg/ml and 10 µg/ml did not affect basal cell proliferation; but in the presence of 55 mM glucose AE reverted the proliferation inhibition exerted by 55 mM glucose by 46%, 34% and 41%, respectively (Fig. 2C). Only 0.1 µg/ml AE reverted the effect of 11 mM glucose by about 30%.

NDGA decreased the basal proliferation at all the concentrations analyzed (Fig. 2D) and could only induce a significant reversal of the effect of 11 mM glucose by 22.7% when used at 3 × 10^−4^ µg/ml (Fig. 2D).Ethanol alone (used as vehicle) assayed at 0.5% final concentration did not affect cell proliferation [proliferation (Abs): 0.64 ±0.04, *n* = 9].

### Effects of AE and NDGA on the production of NO by macrophages under glucose stimulation

**Fig. 3:**
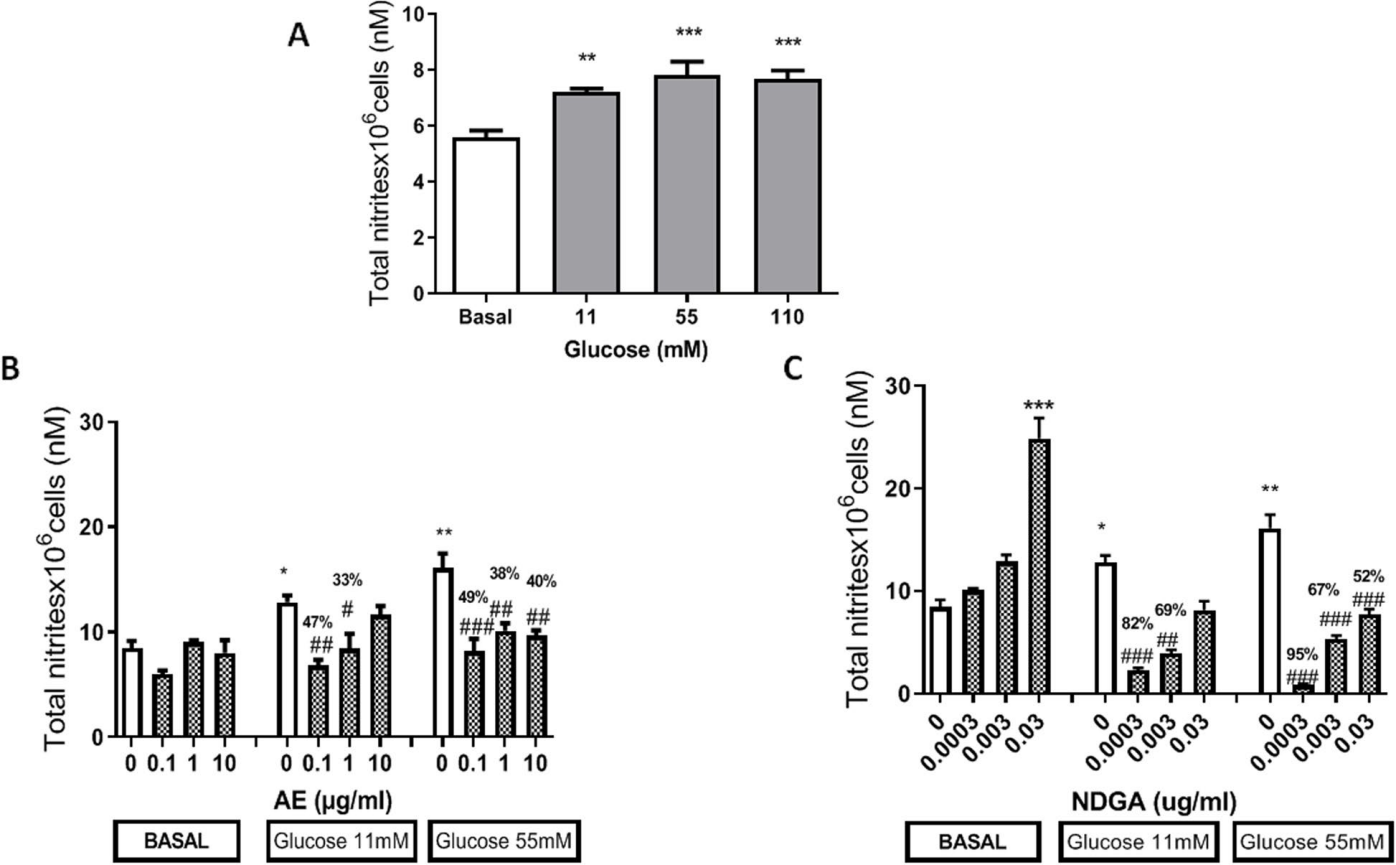
Effect of AE and NDGA on the production of NO by glucose-treated macrophages. **A** Effect of glucose on NO. **B** Effect of AE on NO in glucose-treated cells. **C** Effect of NDGA on the production of NO by glucose-treated cells. Cells were incubated for 24 h in presence of different concentrations of glucose: 5.5 (basal), 11 mM and 55 mM with or without AE or NDGA. Results represent total nitrites x 10^6^ cells (nM) and are expressed as Mean ± SEM of three or more experiments performed in triplicate. The percentage of decrease is also expressed over the bar when necessary.* *p* <0.05, *** *p* < 0.001: significantly different with respect to basal conditions and # *p* < 0.05, ## *p* < 0.01, ### *p* < 0.001: significantly different with respect to each concentration of glucose (One way ANOVA followed by Dunnett’s test)

Taking into account that NO is involved in the modulation of cell proliferation and that it is also produced under inflammatory conditions, the effects of AE and NDGA were studied on the production of this mediator in either the presence or the absence of glucose. Glucose significantly increased the production of NO measured as TN at all concentrations assayed (Fig. 3A).

AE alone did not modify the basal production of NO at any concentration, but could decrease the production of NO induced by 11mM glucose when used at 0.1 µg/ml and 1 µg/ml (around 47% and 32%)and the effect of glucose 55 mM at all concentrations assayed (around 49%, 38% and 40%, respectively, Fig. 3B).

NDGA increased the basal production of NO when used at 0.03 µg/ml (200%), while 0.0003 µg/ml and 0.003 µg/ml reverted the effects of 11 mM glucose (around 82%-69%, respectively).All NDGA concentrations assayed reverted the increase of TN induced by 55 mM glucose (around 95%, 67% and 52%, respectively, Fig. 3C). The effects exerted by AE and NDGA were inversely proportional to their concentration (Fig. 3B and C). Ethanol alone (used as vehicle) assayed at 0.5% final concentration did not affect TN levels (TN x 10^6^ cells: 5.3 ± 0.2nM *n* = 9).

### Effects of AE and NDGA on the production of ROS under the oxidative stress induced by glucose

**Fig. 4:**
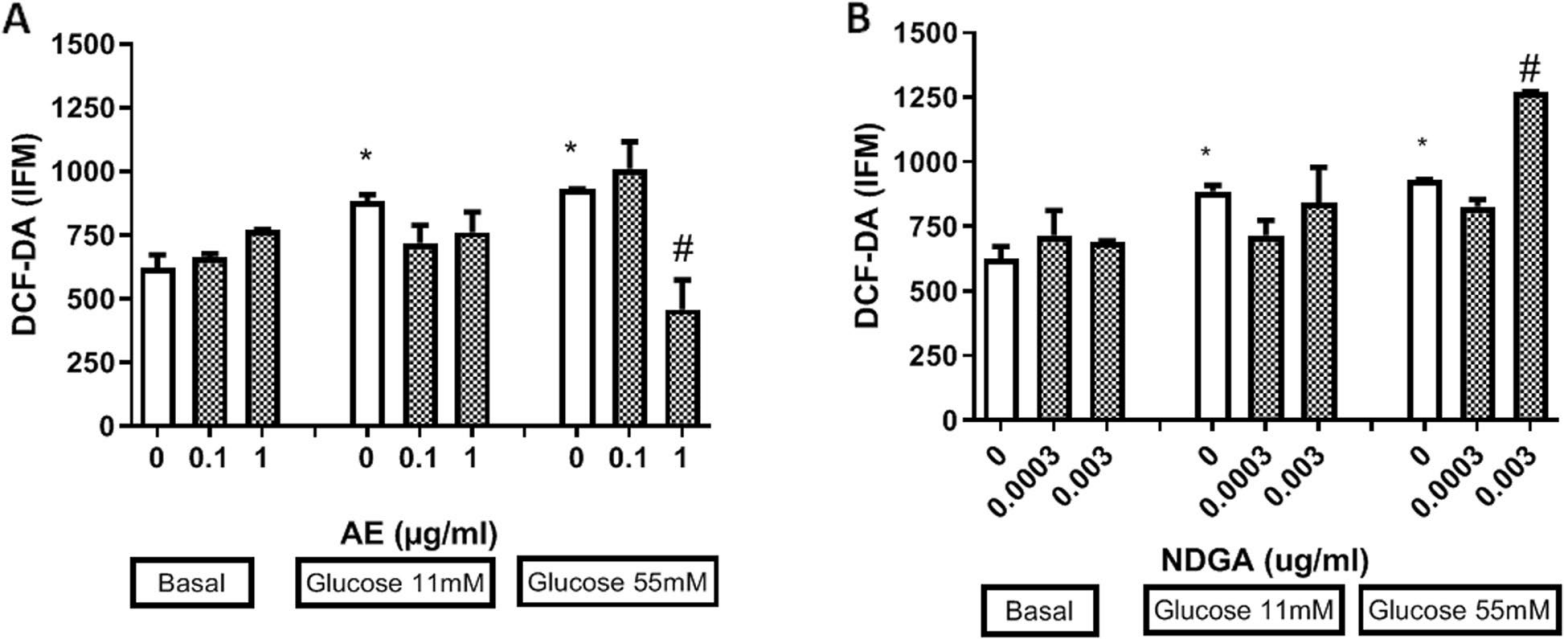
Effect of AE (**A**) and NDGA (**B**) on the production of reactive oxygen species (ROS) by glucose-treated macrophages. Cells were incubated for 24 h in presence of glucose: 5.5 mM (basal), 11 mM and 55 mM with or without AE or NDGA. Results are expressed as Mean ± SEM of three or more experiments performed in triplicate. *p < 0.05 significantly different with respect to basal conditions and # *p* < 0.05: significantly different with respect to each glucose concentration (One way ANOVA followed by Dunnett’s test)

To determine the participation of ROS in the decrease of the proliferation induced by glucose, the effect of AE and NDGA on the production of ROS was studied in either the absence or the presence of glucose. Glucose increased the production of ROS at 11 and 55 mM (Fig. 4 A and B). AE at 1 µg/ml induced a significant reversal of the effect of 55 mM glucose, while 0.003 µg/ml NDGA significantly enhanced the effect of 55 mM glucose (Fig. 4A and B). The other concentrations of AE and NDGA did not produce any changes. Ethanol alone (used as vehicle) assayed at 0.5% final concentration did not affect ROS production (DCF-DA (IFM): 610 ±40, *n* = 9).

### Effects of AE and NDGA on the production of TBARS under oxidative stress induced by glucose

**Fig. 5:**
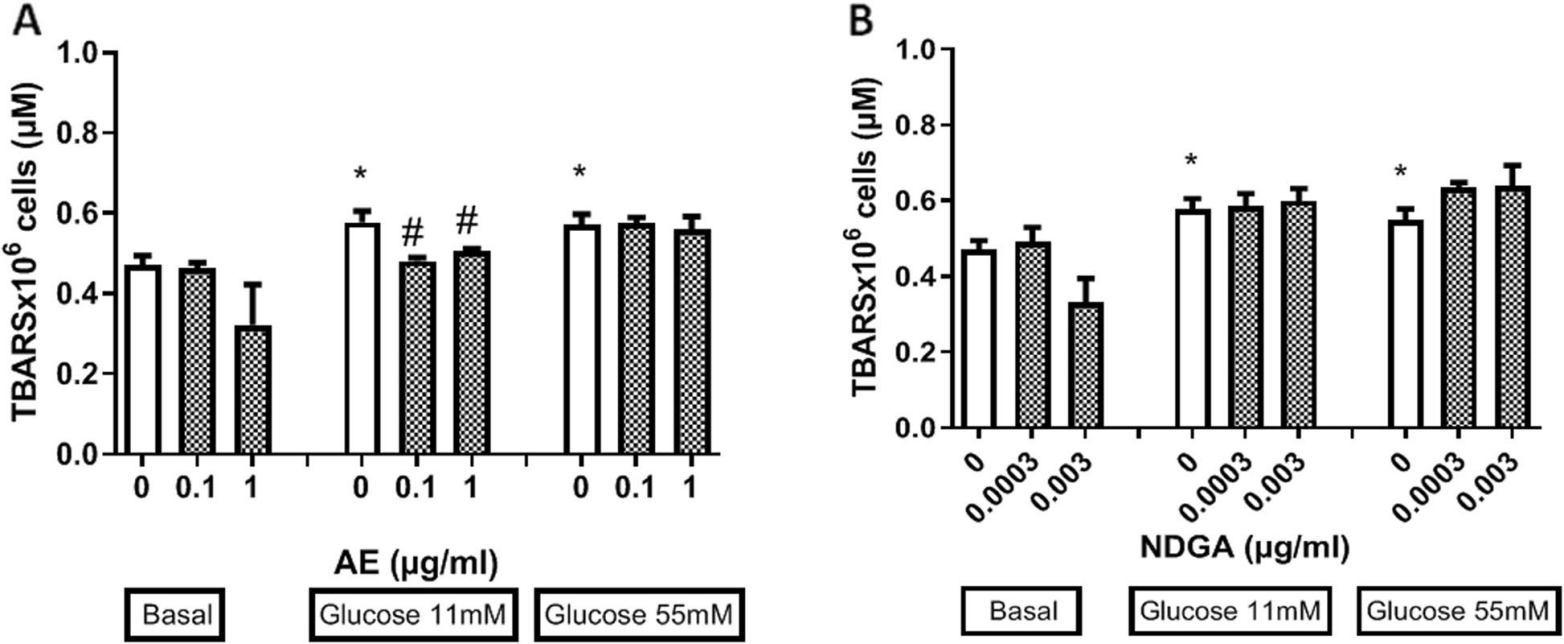
Effect of AE (**A**) and NDGA (**B**) on the production of thiobarbituric acid reactive substances (TBARS) by glucose-treated macrophages. Cells were incubated 24 h in presence of glucose: 5.5 mM (basal), 11 mM and 55 mM with or without AE or NDGA. Results are expressed as Mean ± SEM of three or more experiments performed in triplicate. *p < 0.05: significantly different with respect to basal conditions, # *p* < 0.05, significantly different with respect to each glucose concentration (One way ANOVA followed by Dunnett’s test)

It is known that the increase of TBARS levels is a marker of phospholipids’ peroxidation that can affect the integrity of the cell membrane and cell proliferation. Therefore, the effects of AE and NDGA on the production of TBARS were studied in either the absence or presence of glucose.At11mM and 55 mM glucose significantly increased the production of TBARS (Fig. 5A and B). AE, at the two concentrations assayed (0.1 and 1 µg/ml), could only revert the effect of 11 mM glucose (around 17% and 14% respectively) (Fig. 5A and B).NDGA did not modify the effects exerted by glucose at any concentration employed. Ethanol alone (employed as vehicle) assayed at 0.5% final concentration did not affect the production of TBARS (TBARSx 10^6^ cells: 0.39 ±0.04 µM, *n* = 9).

### Effects of AE and NDGA on the production of TNF-αunder the oxidative stress induced by glucose

TNF-α is a pro-inflammatory cytokine that can promote the release of NO and affect cell proliferation. Thus, the effect of AE and NDGA on the production of TNF-αin either the absence or the presence of glucose was studied.

**Fig. 6:**
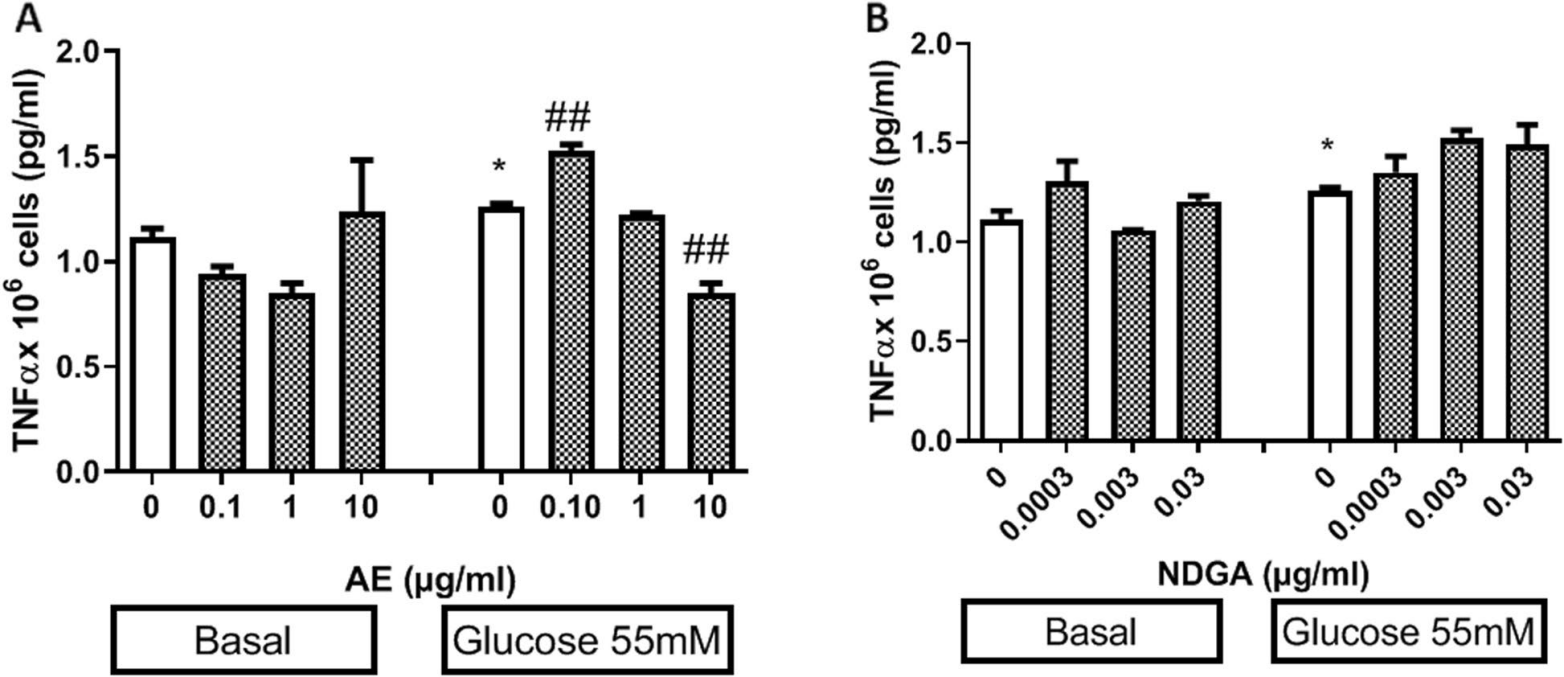
Effect AE (**A**) and NDGA (**B**) on the production of tumor necrosis factor (TNF-α) in glucose-treated macrophages. Cells were incubated 24 h in the presence of glucose 5.5 mM (basal) and 55 mM with or without AE or NDGA. Results are expressed as Mean ± SEM of three or more experiments performed in triplicate. *p <0.05: significantly different with respect to basal conditions and #*p* < 0.05, ##*p* < 0.01: significantly different with respect to each glucose concentration (One way ANOVA followed by Dunnett’s test)

Only 55 mM glucose caused an increase in TNF-α levels (Fig. 6A and B).A low concentration of AE enhanced this effect while higher concentrations reverted it by 33% (Fig. 6 A). NDGA did not exert a significant effect on the production of TNF-αunder neither basal conditions nor in presence of glucose (Fig. 6B). Ethanol alone (used as vehicle) assayed at 0.5% final concentration did not affect the release of TNF-α (TNF-α x 10^6^ cells: 1.15 ±0.03pg/ml, *n* = 9).

### Effects of AE and NDGA on reduced gluthatione (GSH) levels under the oxidative stress induced by glucose

**Fig. 7:**
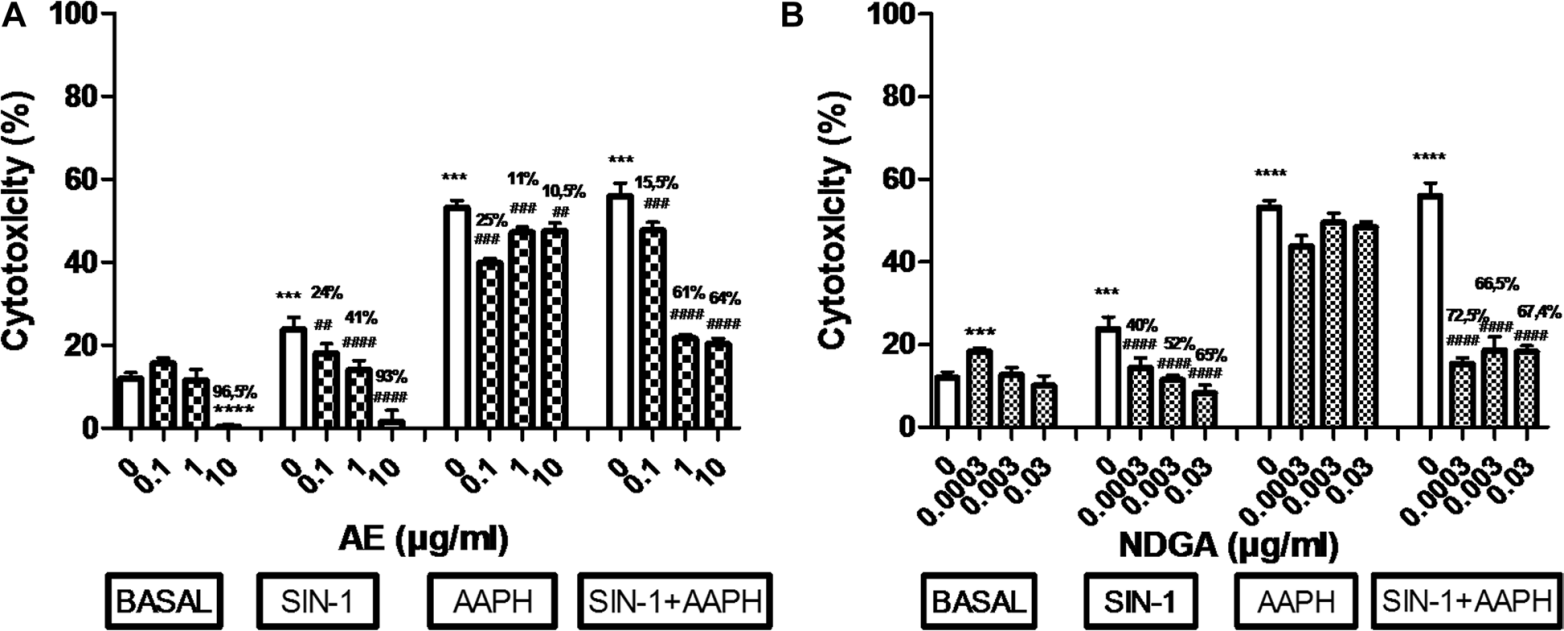
Effect of AE (**A**) and NDGA (**B**) on the production of reduced glutathione (GSH) by glucose-treated macrophages. Cells were incubated for 24 h in the presence of glucose 5.5 mM (basal), 11 mM and 55 mM with or without AE or NDGA. Results are expressed as Mean ± SEM of three or more experiments performed in triplicate. *p* < 0.05: significantly different with respect to basal conditions and #*p* < 0.05, ##*p* < 0.01, ###*p* < 0.001: significantly different with respect to each glucose concentration (One way ANOVA followed by Dunnett’s test)

Since reduced glutathione is an antioxidant molecule that is found at low levels during oxidative stress, the effect of AE and NDGA on the levels of this mediator were studied in either the absence or the presence of glucose. Figure 7A and B show that glucose decreased GSH levels significantly only at 11mM. Under basal conditions, AE increased GSH levels at both concentrations assayed (97%, 51% for both concentrations of AE, respectively). In the presence of 11 mM and 55 mM glucose, AE induced an increase of GSH only at the lowest concentration (around 65% and 24%, for 11 mM and 55 mM, respectively, Fig. 7A). NDGA did not modify the basal GSH production but it reverted the effect of 11 mM glucose at both concentrations tested (68% and 85%, for each NDGA concentration, respectively).Only the higher NDGA concentration reverted the effect of 55 mM glucose (71%).Ethanol alone (used as vehicle) assayed at 0.5% final concentration did not affect the production of GSH (GSH x 10^6^ cells: 150 ±12µM, *n* = 9).

### Effects of AE and NDGA on the cytotoxicity induced by SIN-1 and AAPH in macrophages

To confirm the effect of AE and NDGA on the production of NO and ROS, their effect on the abrogation of cytotoxicity was studied in the presence of SIN-1,a NO generator and AAPH, a ROS generator, and in presence of a combination of both.

**Fig. 8:**
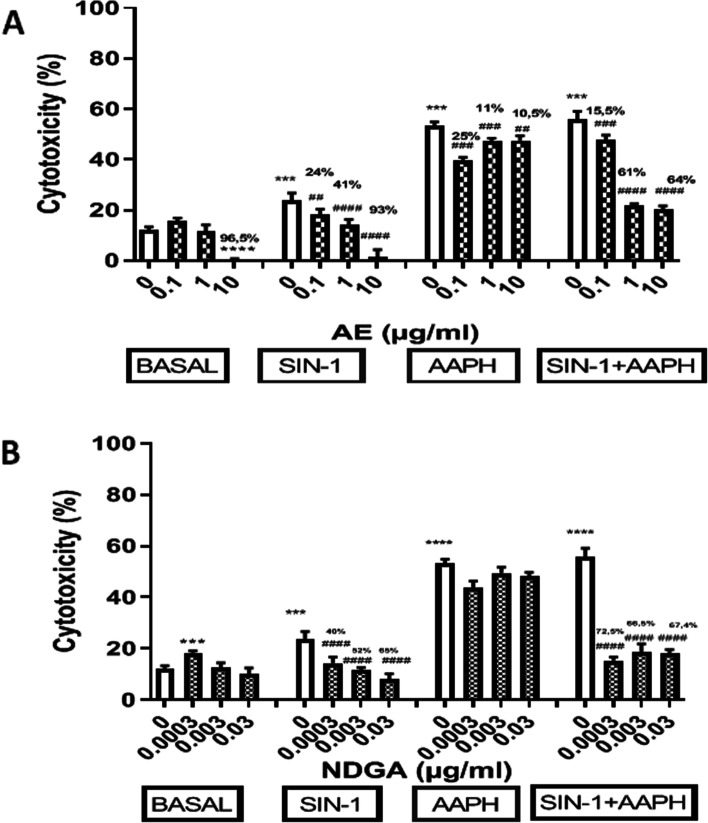
Effect of AE (**A**) and NDGA (**B**) on cytotoxicity in SIN-1, AAPH, SIN-1 + AAPH-treated macrophages. Cells were incubated 24 h in either the absence or the presence of 20 µM SIN-1, 12.5 µM AAPH or both with or without AE or NDGA. Results are expressed as Mean ± SEM of three or more experiments performed in triplicate. ***p < 0.001, ****p < 0.0001: significantly different with respect to basal conditions and ##*p* < 0.01, ###*p* < 0.001, ####*p* < 0.0001: significantly different with respect to each glucose concentration (One way ANOVA followed by Dunnett’s test)

SIN-1, AAPH and their combination increased cell toxicity (around 100%, 340% and 360% respectively, Fig. 8A and B). The toxicity induced by AAPH was 3.4 times greater than that produced by SIN-1. The highest AE concentration (10 µg/ml) decreased basal cell toxicity by 95% and abrogated the effect of SIN-1 and AAPH at all concentrations assayed (Fig. 8A). NDGA increased the basal cytotoxicity at low concentration but could revert the effect of SIN-1 or the combination SIN-1+ AAPH at different % in relation with its concentration (Fig. 8B).Ethanol alone (used as vehicle) assayed at 0.5% final concentration did not affect cell cytotoxicity (cytotoxicity: 10 ±0.30%, *n* = 9).

### Protective effects of AE and NDGA against induced oxidative and nitrosative stress on HDL and LDL

#### Effects of AE and NDGA on the antioxidant activity of HDL PON- 1 under nitrosative and oxidative conditions

**Fig. 9:**
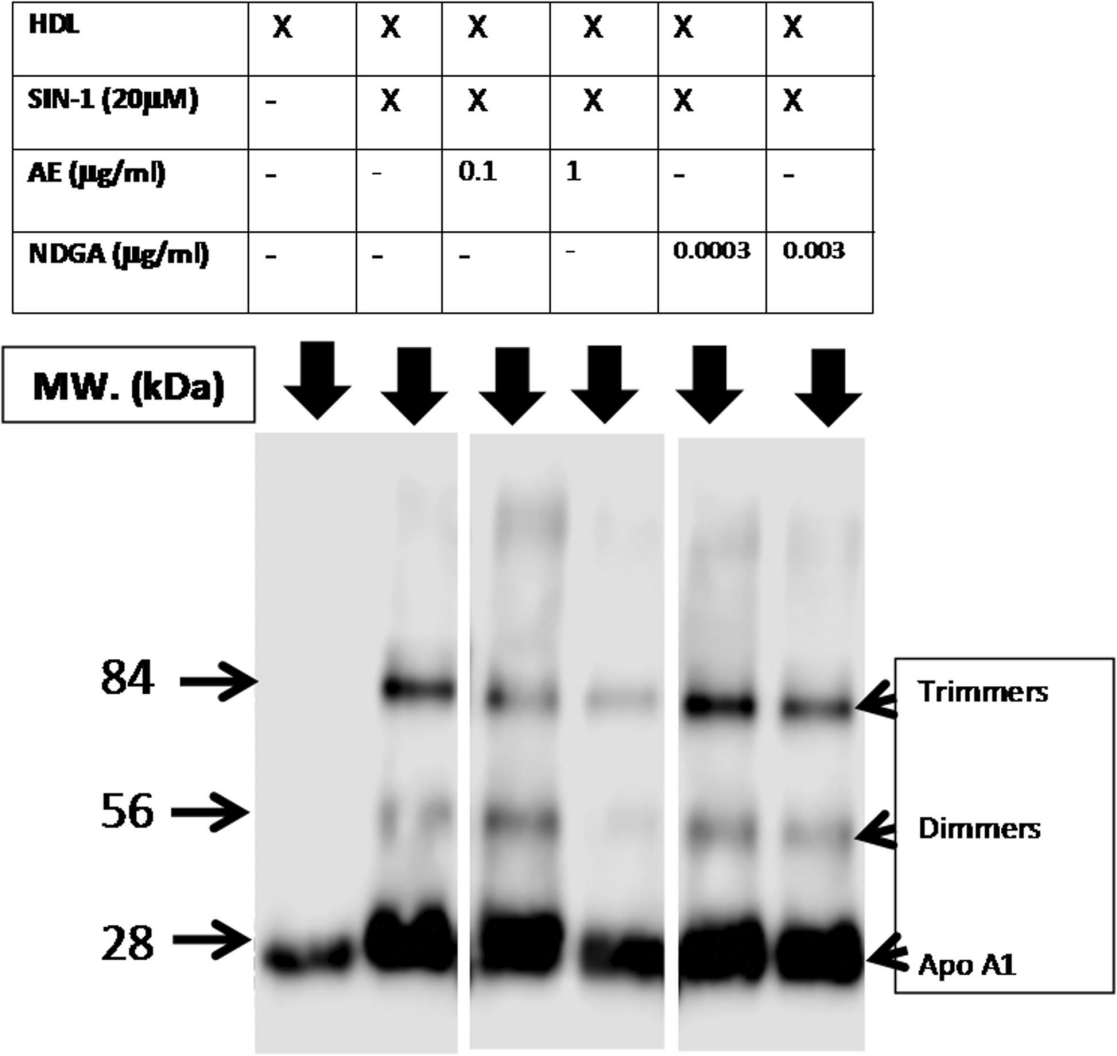
Protective effect of AE and NDGA activity against induced oxidative (**A**) and nitrosative (**B**) stress on HDL-PON-1. HDL was incubated in either the presence or absence of 5 mM AAPH or 10 µM SIN-1 with or without AE or NDGA.PON-1 activity was determined by the capacity of the enzyme to hydrolyze phenylacetate. Results are expressed as Mean ± SEM of three or more experiments performed in triplicate. **p < 0.01 significantly different with respect to basal conditions and #p < 0.05, ##p < 0.01: significantly different with respect to AAPH (**A**) or SIN-1 (**B**) (One way ANOVA followed by Dunnett’s test)

As PON-1plays a major role in the prevention of LDL oxidation, the effects of AE and NDGA were studied on the activity of PON-1under nitrosative and oxidative stress conditions. AAPH induced a significant decrease inPON-1 activity, and this effect was reverted by neither AE (0.1, 1, 10 µg/ml) nor NDGA (0.0003, 0.003, 0.03 µg/ml, Fig. 9 A). On the other hand, SIN-1decreased PON-1 activity, but low concentrations of AE and NDGA were able to revert this effect around 13% and 27% (Fig. 9B).

#### Effect of AE and NDGA on the structure of HDL under the nitrosative stress induced by SIN-1

**Fig. 10:**
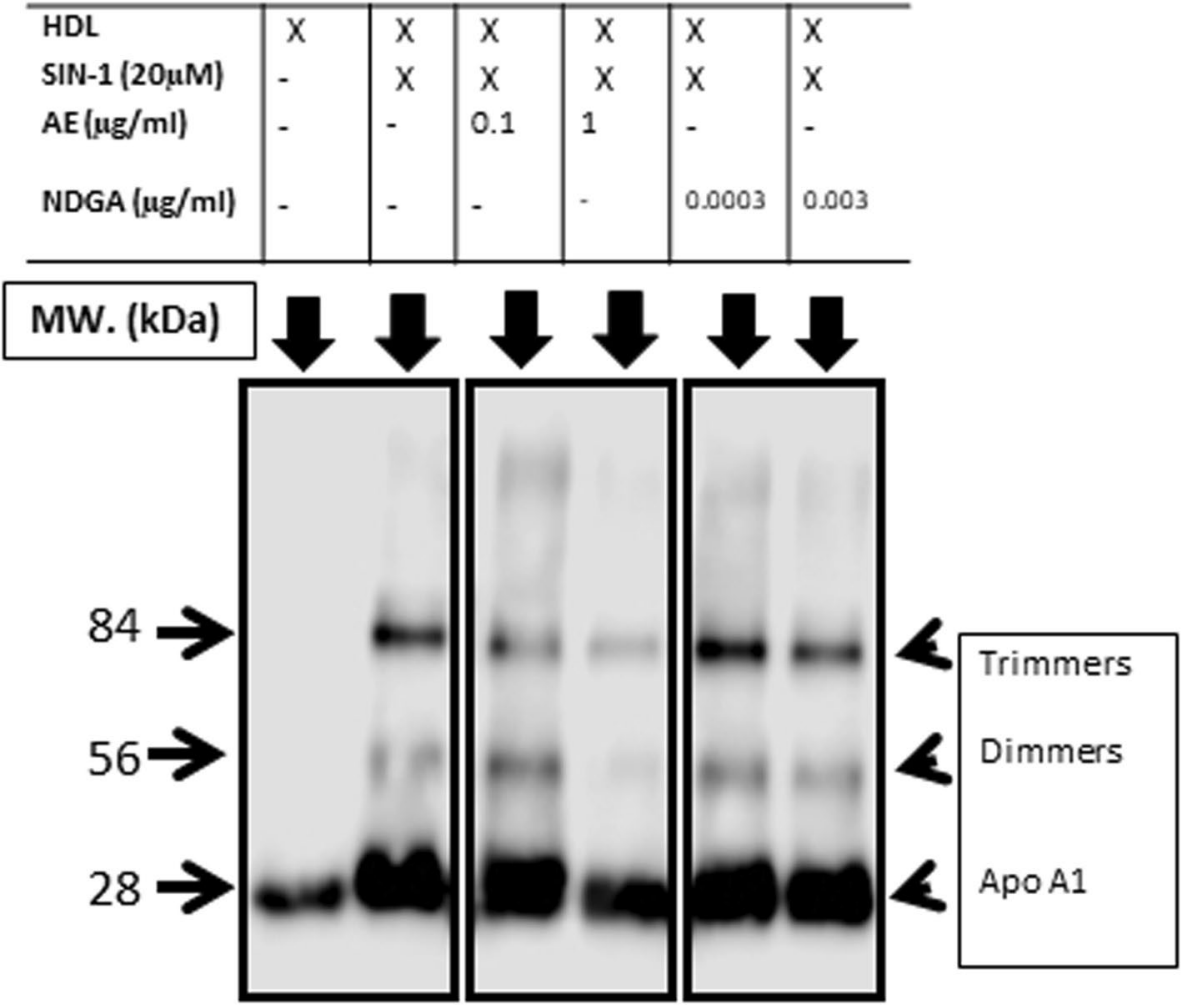
Effect of AE and NDGA on the structure of HDL subjected to nitrosativestress. HDL was incubated either alone or with 20 µM SIN-1 in either the presence or absence of AE (0.1 µg/ml and 1 µg/ml) or NDGA (0.0003 µg/ml and 0.003 µg/ml).For detection of ApoA1, an HRP-conjugated antibody (anti-Apo-A1 (HRP), 1:1,000 in 10% FCS) was used. Blots were then incubated with the ECL PLUS reagent (Amersham), developed and scanned in a chemiluminescent scanner (LICOR C-DIG it, LICOR Biosciences) and quantified with the Image Studio software. Representative westernblot of fractionated samples.To improve clarity the the cropped blots were used in the figure. The samples of fractionations were derived from the same experiment and the blots were run in parallel.Full-length blot is shown in Supplementary file

To determine the mechanism of the decrease of PON-1 activity induced by SIN-1, the structure of HDL was studied by analyzing ApoA1 by westernblot. SIN-1 modified the electrophoretic profile of HDL, which formed dimers and trimers upon treatment with the pro-oxidant substance. The degradation of HDL was attenuated by the treatment with AE at both concentrations assayed, but the higher effect was observed with 1 µg/ml. NDGA could only prevent degradation at the highest concentration assayed (0.003 µg/ml) (Fig. 10).

### Effect of AE and NDGA on LDL under Cu^2+^-induced oxidative stress

**Fig. 11:**
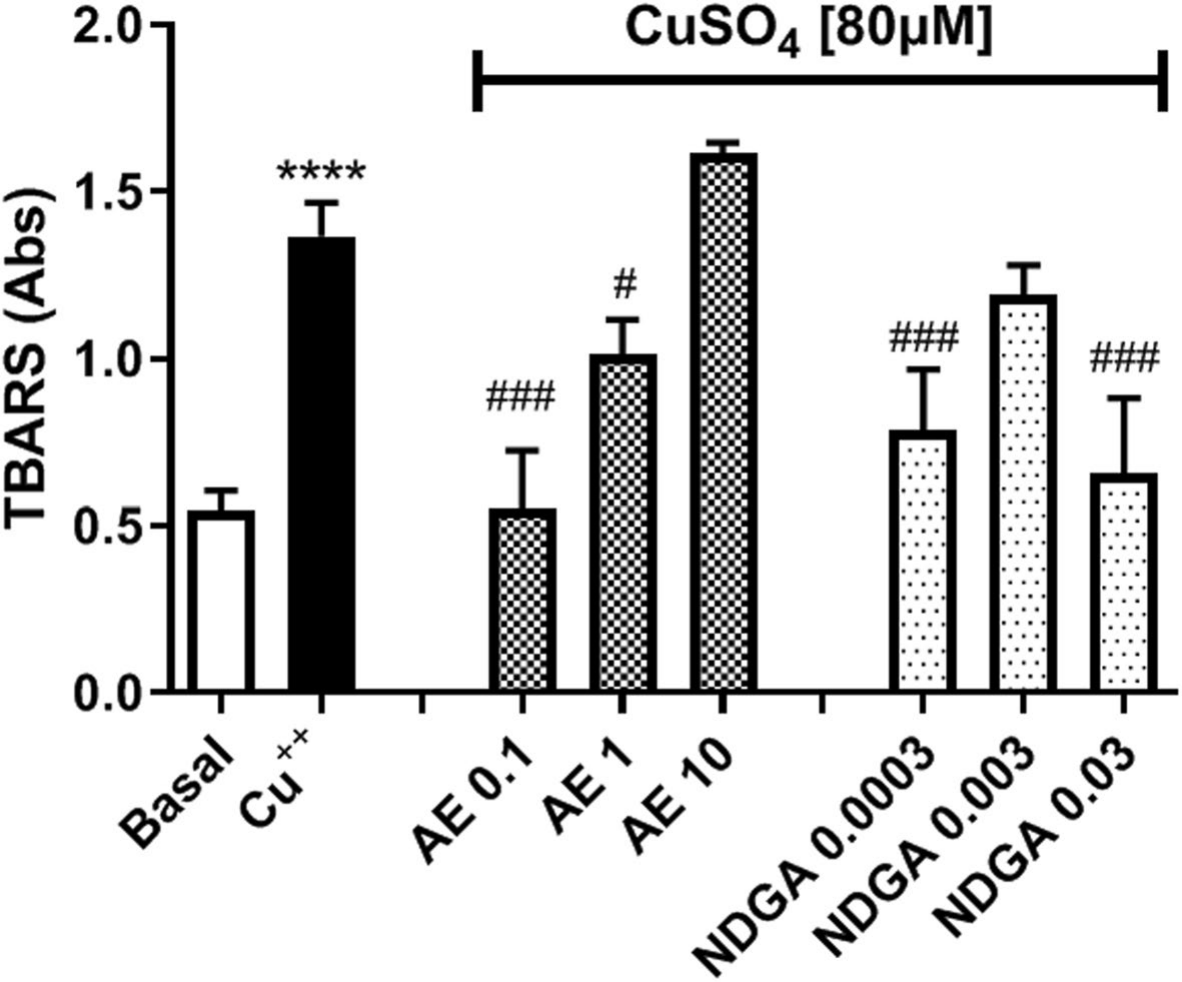
Effect of AE and NDGA on the generation of TBARS in LDL subjected to Cu^2+^-induced oxidation. LDL was incubated either alone or in the presence of Cu^2+^ with or without AE or NDGA. Results are expressed as the Mean ± SEM of three or more experiments performed in triplicate. ****p < 0.0001 significantly different with respect to basal conditions and #p < 0.05, ## *p* < 0.01, ### *p *<  0.001: significantly different with respect to the treatment with Cu^2+^ (One way ANOVA followed by Dunnett’s test)

To confirm the antioxidant activity of AE and NDGA, LDL was subjected to oxidative stress induced by CuSO_4,_ and the production of TBARS was analyzed.CuSO_4_increasedTBARSlevels significantly (154%, Fig. 11) and AE reverted this phenomenon at 0.1 µg/ml and 1 µg/ml. The reversal was maximum at the lowest AE concentration. NDGA reverted the effect only at 0.0003 µg/ml and 0.03 µg/ml (Fig. 11).

## Discussion

This work demonstrates the *in vitro* antioxidant and anti-inflammatory activities of the aqueous extract of *Larrea divaricata* on macrophages under oxidative stress induced by glucose. The extract also protected human HDL and LDL from the oxidative and nitrosative stress induced by AAPH and SIN-1, respectively. NDGA appeared to be involved in the antioxidant action of the AE, mainly under nitrosative stress conditions, while other compounds could be involved on the prevention of both nitrosative and oxidative stress.

The diagnostic criterion for diabetes is a blood glucose concentration of >100 mg/dl. Therefore, in the present study, 5.5 mM glucose (100 mg/dl) was used as control, and 11mM and 55 mM glucose were used as high-glucose levels to emulate diabetic status in macrophages.

In relation to the model used, results demonstrated that glucose, at 11 and 55 mM, decreased the proliferation and viability of macrophages in direct relation to oxidative and nitrosative stress and to inflammatory status by inducing NO, ROS and TNF-α respectively. These events induced lipid peroxidation and a decrease in GSH.

Glucose induced an increase of TNF-α levels, with this effect being stronger at 55 mM, the concentration at which the highest NO production and the strongest inhibition of cell proliferation were observed. It is known that, the production of NO can be related to the induction of TNF-α, which is generated during inflammatory processes and that nitrosative and oxidative stress induce a decrease in cell proliferation and apoptosis [21–23].In concordance with our results, other authors have observed that elevated glucose levels in the culture medium decreases the proliferation of fibroblasts, endothelial cells, keratinocytes and mesenchymal stem cells by oxidative stress [24–27]. Glucose also, increased lipid peroxidation, at the same time that decreased glutathione, at the two concentrations tested, showing a direct relation to the increase of ROS and NO levels. The capacity of oxidative and nitrosative stress to decrease cell proliferation through lipid peroxidation, is related to a decreased in reduced glutathione, observed in other models [18, 28].

Under this model, the extract prevented the decrease on cell proliferation induced by glucose with an effect stronger than that exerted by NDGA. Also, a good correlation between NO levels and cell proliferation was observed, e.g., in the cases in which AE did not induce any change in the NO levels, no variations in the cell proliferation rate at basal status were observed. Conversely, when AE reverted the effects of glucose on NO release, it also reverted the effect on cell proliferation. The same phenomenon was observed for NDGA, suggesting that NDGA played a major role in the overall effect exerted by the whole extract.

AE also reverted the effects of glucose on the production of ROS, on induction of lipid peroxidation (both effects independent on NDGA) and on the decrease of GHS, related to NDGA presence. It can be said that compounds other than NDGA could participate in the effects observed for the whole extract. Besides, it was demonstrated that AE, in presence of glucose, could modulate the level of NO by modulating TNF-α level, principally at high concentrations. The effect of NDGA on NO appeared to be independent on TNF-α as this compound did not revert TNF-α level exerted by glucose.

The antioxidant properties of AE have previously been reported. In fact, AE can modulate the production of H_2_O_2_ and NO by exerting CAT, Px and SOD-like activities, but also through its capacity to modulate the level of these antioxidant enzymes, as demonstrated in rat submandibular glands subjected to oxidative stress induced by the administration of streptozotocin [8].

To confirm the effect of AE and NDGA on oxidative and nitrosative stress, macrophages were treated with the peroxinitrite generator SIN-1 and the ROS generator AAPH. In aqueous solution SIN-1 generates NO and O_2_^−^. [29, 30], which combine to form the peroxynitrite anion (OONO^−^.) [31]. On the other hand, AAPH, is a generator of free radicals involved in lipid peroxidation. AAPH produces nitrogen and carbon radicals, which can react with molecular oxygen to generate the peroxylradical. Thus, APPH would act as a lipid peroxidation inducer [32]. In this model, both generators induced cytotoxicity on macrophages but the effect observed with AAPH was stronger than that of SIN-1. Again, and unlike AE, NDGA proved to exert a cytotoxic effect under basal conditions. All the AE concentrations assayed and the relative concentration of NDGA prevented SIN-1-induced cytotoxicity, though in different degrees. The participation of NDGA in this response has been suggested, as NDGA presents peroxinitrite scavenger activity, prevents nitration of proteins and nitrosative stress-induced nephrotoxicity [33].

Unlike NDGA, AE reverted the cytotoxicity induced by AAPH, suggesting that other antioxidant compounds may be present in the extract. The capacity of AE to prevent oxidative stress can be attributed to a SOD-like activity, which is known to be low for NDGA [34].

When the combination of ROS and RNS generators was used, the reversal of the effect exerted by AE and NDGA was marked, causing a reduction of cytotoxicity levels from 60% to15%-20% (basal levels). In this case, the activity of NDGA was stronger than that of AE at the lowest concentration. The strong effect of AE and NDGA in cells treated with a combination of oxidative and nitrosative stress generators may be attributed to the O_2_.^−^ scavenging capacity of AE and NDGA exerted through SOD, CAT and Px activities. Thus, the amount of O_2_.^−^to produce peroxinitrites by SIN-1 is considerably reduced.

Taking into account that the oxidation of LDL and the loss of PON-1 antioxidant activity, caused by oxidative stress, are related to atherosclerosis, and that AE and NDGA presented antioxidant activity and prevented lipid peroxidation in macrophages, the effects of AE and NDGA were also studied on HDL and LDL particles subjected to oxidative stress induced by SIN-1 and AAPH.

Since the quantitative determination of HDL-associated cholesterol is insufficient to assess the anti-atherogenic activity of this lipoprotein, its functionality and structure were evaluated in this work. AAPH and SIN-1 decreased PON-1 activity, but AE and NDGA could only revert the effect exerted by SIN-1.NDGA played a key role in the effect exerted by the extract. The PON-1 protective activity of plant extracts has been reported by other authors [35, 36].

The loss of the antioxidant activity of HDL apoproteinase, consequence of the nitrosative effect exerted by SIN-1, was then studied. Western blot experiments revealed a widening of the 28 kDa band (Apo-A1) in the presence of SIN-1, as compared to purified HDL. Furthermore, the appearance of two new bands of 56 and 84 kDa suggested the aggregation of Apo-A1 in dimers and trimers. When HDL was incubated with SIN-1in the presence of AE, a marked decrease in the intensity of these bands was observed, demonstrating that AE also protected the structure of HDL by preventing ApoA1 aggregation. Although NDGA also reduced band intensity, its effect was weaker than that of AE, suggesting that the action of the extract was due to the synergistic effect between NDGA and other compounds.

Following this reasoning, the effect of AE and NDGA on copper ion-induced LDL oxidation was analyzed [37]. Cu^2+^ is known to remove unpaired electrons from unsaturated fatty acids from LDL, generating a chain reaction that ultimately causes the formation of malondialdehyde that is detected in the TBARS assay. Cu^2+^increased LDL TBARS and AE reverted this effect in an inverse relationship with its concentration. NDGA reverted the effect of Cu^2+^at the highest and lowest concentrations. Therefore, it can be inferred that NDGA would be involved in the antioxidant activity observed with the lowest concentration of the extract and that there were compounds in the extract that antagonized the effect of NDGA at high AE concentrations.

The protective effect of AE on PON-1 activity and on Cu^2+^-induced oxidation of LDL could be related to the presence of NDGA, at least at low AE concentrations. The preventive effect of NDGA could be attributed to its NO and peroxinitrite scavenger activity, as demonstrated elsewhere [33]. Moreover, the protective effect of AE on the oxidation of LDL induced by Cu^2+^could be related to the inhibition of lipid peroxidation, free radical scavenger activity and metal reducing power exerted by AE and NDGA [34, 38].

Finally, the overall antioxidant activity of AE could be related to the presence of other polyphenolic compounds different from NDGA. A previous The HPLC-UV analysis of AE demonstrated the presence of polyphenols such as the lignin NDGA (retention time: 47 min), the phenolic acid 4-HBA (retention time: 12 min), and the flavonoid rutin(retention time: 31.6 min). Flavonoids such as epicatechinandquercetin-3-O-arabinopyranoside were also detected by HPLC-MS/MS (data not shown). The latter compounds have a very well-documented antioxidant activity associated with their capacity to reduce metals like iron and to modulate the enzymatic antioxidant system [39].

## Conclusions

AE protected macrophages from the oxidative and nitrosative effects induced by glucose, demonstrating its antioxidant and anti-inflammatory properties. AE also protected the function and structure of HDL from nitration and was capable of inhibiting the Cu^2+−^induced LDL oxidation.

The results obtained herein are promising and encourage us to continue with *in vivo* studies to determine if the whole extract could be used as an adjuvant phytotherapyin the prevention or treatment of atherogenic complications in diabetic patients.

## Supplementary Information

**Additional file 1.**

## Data Availability

Data used to support the findings of this study are included in the article.

## Abbreviations

4-HBA: 4-hydroxybenzoic acid
AAPH: 2,2’-azobis (2-amidinopropane) dihydrochlorohydrate
AE: aqueous extract
Apo-B: apolipoprotein B
CAT: catalase
DM: diabetes mellitus
DTNB: 5.5´-dithio-bis-(2-nitrobenzoic acid)
EDTA: ethylenediaminetetraacetic acid
GSH: reduced glutathione
HDL: high density lipoprotein
LDL: low density lipoprotein
MFI: mean population cell fluorescence intensity
MTT: 3- (4,5-dimethylthiazol-2-yl) -2,5-diphenyltetrazolium bromide
NDGA: nordihydroguaiaretic acid
NO: nitric oxide
ox-LDL: oxidized low density lipoprotein
PON 1: paraoxonase 1
Px: peroxidase
RNS: reactive nitrogen species
ROS: reactive oxygen species
SIN-1: 3-morpholinosydnonimine
TBARS: thiobarbituric acid reactive substances
TN: total nitrites
TNF-α: tumor necrosis factor-α

## Declarations

Blood was obtained from eight healthy and normolipemic volunteers. Informed consent was obtained from all volunteers and the research was carried out in accordance with the principles of the Declaration of Helsinki. These tests were carried out at the Glycation, Oxidation and Disease Laboratory, Touro University of California, University of Touro, California, USA. All human experimental protocols were approved by the Ethics committee of University of Touro, California.
