# Correction: *Larrea divaricata*: anti-inflammatory and antioxidant effects on macrophages and low density lipoproteins

**DOI:** 10.1186/s12906-022-03621-1

**Published:** 2022-07-08

**Authors:** Ignacio Peralta, Carla Marrassini, Malen Saint Martin, Laura Cogoi, Maria Rosario Alonso, Alejandro Gugliucci, Claudia Anesini

**Affiliations:** 1 Universidad de Buenos Aires, Consejo Nacional de Investigaciones Cientificas y Tecnicas (CONICET), Instituto de Quimica y Metabolismo del Farmaco (IQUIMEFA), Buenos Aires, Argentina; 2 Cátedra de Farmacognosia, Facultad de farmacia y Bioquimica, Universidad de Buenos Aires, Buenos Aires, Argentina; 3 Disease Laboratory, Touro University of California, University of Touro, Vallejo, CA USA

**Correction: BMC Complement Med Ther 22, 84 (2022)**

**https://doi.org/10.1186/s12906-022-03547-8**

Following publication of the original article [1], the authors reported errors in the proof.

In the second paragraph of Background section, highlighted in **bold** was removed.

The sentence currently reads:

Since the antioxidant systems may sometimes be insufficient, the administration of exogenous innocuous antioxidant substances may be required to avoid the diabetic complications. The current trend is to search for antioxidant substances with the capacity to modulate blood lipid levels. **These substances may be found in plant extracts, which have been consumed for generations and generally are being well tolerated y people possess which present fewer adverse effects than synthetic compounds.** These substances may be found in plant extracts, which have been consumed for generations and, when used in a correct dose, were generally better tolerated than synthetic compounds.

The sentence should read:

Since the antioxidant systems may sometimes be insufficient, the administration of exogenous innocuous antioxidant substances may be required to avoid the diabetic complications. The current trend is to search for antioxidant substances with the capacity to modulate blood lipid levels. These substances may be found in plant extracts, which have been consumed for generations and, when used in a correct dose, were generally better tolerated than synthetic compounds.

The name of the funder was changed and the change have been highlighted in **bold typeface**.

Funding

This work was supported by **PIP 00067 CO CONICET** and UBACYT from Buenos Aires University 20020130100686BA. The funding body approved the design of the study and allowed authors the acquisition of reagents and all materials used in the experiments.

The correct Figures 7 and 9 are given below.

**Figure 1:**
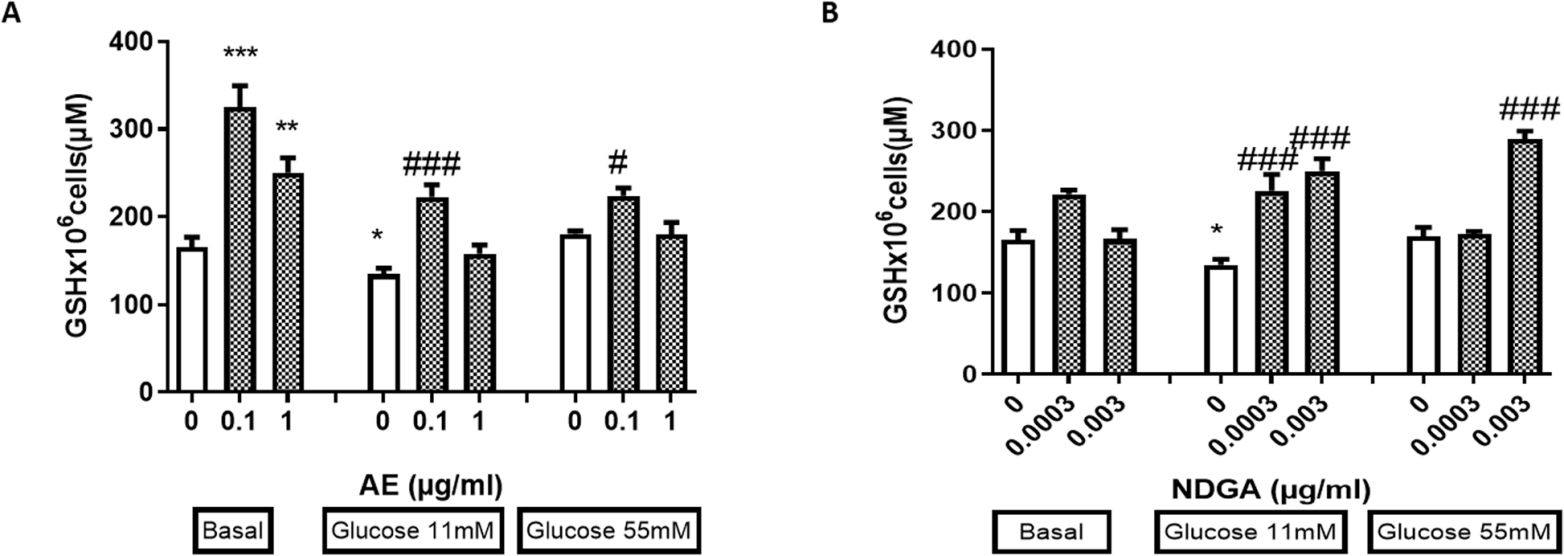
**Fig. 7**

**Figure 2:**
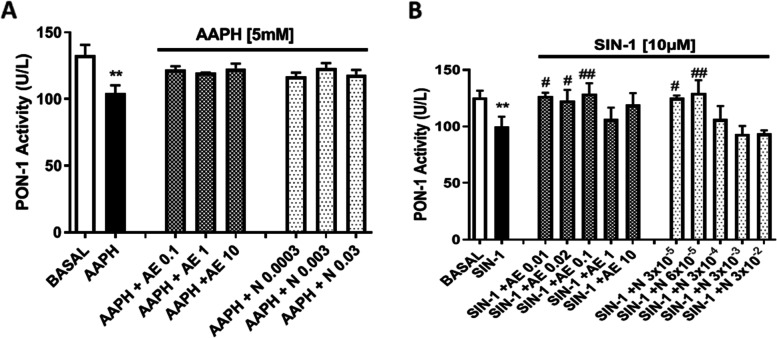
**Fig. 9**

The original article [1] has been updated.
