# Supplementary material for: Larrea divaricata: anti-inflammatory and antioxidant effects of on macrophages and low density lipoproteins
Source: BMC Complement Med Ther. 2022 Mar 23;22:84. doi: 10.1186/s12906-022-03547-8 (PMC8941816; doi:10.1186/s12906-022-03547-8)
Supplement: Supplementary file 1 — Additional file 1. [file 12906_2022_3547_MOESM1_ESM.zip › gel denat HDL vsSIN.pdf]

Image Report: gel denat HDL vsSIN

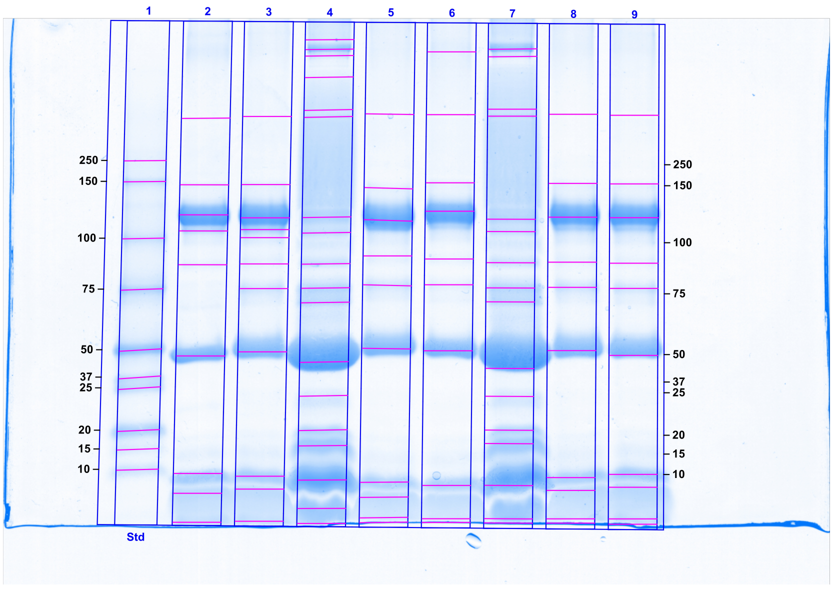

C:\Users\rcaccavello\Documents\NAKO PERALTA\gel denat HDL vsSIN.scn

---

Acquisition Information

Imager	GS-900™
Serial Number	SGCWBA10683
Firmware Version	61
Software Version	6.0.0.25
Application	Colloidal Coomassie
Scan Mode	Transmissive
Scan Color	Red
OD Calibration	Yes
Flat Field	Red

Image Information

Acquisition Date	10/10/2018 3:11:13 PM
User Name	Russell Caccavello
Image Area (mm)	X: 98.0 Y: 69.0
Pixel Size (µm)	X: 63.5 Y: 63.5
Data Range (OD)	0.000 - 2.128

Analysis Settings

Detection	Lane detection:<br>Manually created lanes<br><br>Band detection:<br>Automatically detected bands with sensitivity: High<br>Manually adjusted bands<br><br>Lane Background Subtraction:<br>Lane background subtracted with disk size: 0.1

	Lane width: Variable
Mol. Weight Analysis	Standard: Bio-Rad Precision Plus<br>Standard lanes: first<br>Regression method: Linear (semi-log)

Calibration Report

OD Value	Mean Intensity	Max Value	Min Value	Std Dev
0.00	6709	42437	4526	528.86
0.08	16753	35882	14669	606.78
0.27	32050	42005	30184	479.75
0.46	42867	45882	41147	351.29
0.69	50833	55228	49694	273.86
0.88	56040	57122	55219	179.94
1.13	59275	60060	58639	136.07
1.34	61337	61799	60894	99.34
1.55	62756	63239	62435	75.61
1.75	63608	63843	63325	60.40
1.97	64239	64434	64024	47.34
2.18	64632	64787	64437	39.62
2.39	64890	65027	64732	32.96
2.59	65047	65169	64918	28.18
2.83	65155	65271	65041	25.07
3.07	65227	65332	65126	23.09
3.30	65270	65377	65173	22.06
3.46	65283	65391	65180	22.80
3.64	65287	65412	65183	23.11
3.88	65295	65417	65173	23.61

Note: Values reported are specular OD values to account for differences in how scanner-based instruments measure diffuse and specular samples.

Lane Statistics

Lane No.	Adj. Total Band Vol. (OD)	Total Band Vol. (OD)	Adj. Total Lane Vol. (OD)	Total Lane Vol. (OD)	Bkgd. Vol. (OD)	Norm. Factor
1	1,151.90	2,213.82	1,202.67	3,433.90	2,231.22	N/A
2	1,200.29	3,584.22	1,251.64	4,756.95	3,505.31	N/A
3	2,510.09	4,073.15	2,645.16	5,331.29	2,686.13	N/A
4	4,158.00	7,767.70	4,226.87	10,628.61	6,401.74	N/A
5	2,991.29	4,123.33	3,028.40	5,102.27	2,073.87	N/A
6	1,254.55	3,673.21	1,288.99	4,654.54	3,365.54	N/A
7	1,359.99	7,864.52	1,399.11	10,416.80	9,017.69	N/A
8	2,787.53	4,106.46	2,912.66	5,258.35	2,345.69	N/A
9	2,036.84	4,462.49	2,241.99	5,664.36	3,422.37	N/A

Lane And Band Analysis

Lane 1 - Bio-Rad Precision Plus

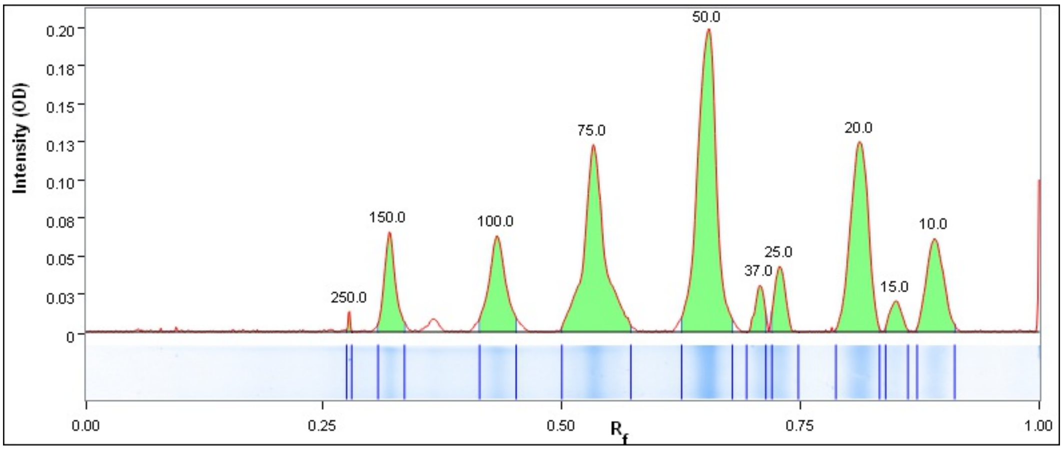

Band No.	Band Label	Mol. Wt. (KDa)	Relative Front	Adj. Volume (OD)	Volume (OD)	Abs. Quant.	Rel. Quant.	Band %	Lane %
1		250.0	0.278	2.74	23.08	N/A	N/A	0.2	0.2
2		150.0	0.319	67.31	151.91	N/A	N/A	5.8	5.6
3		100.0	0.432	98.09	212.02	N/A	N/A	8.5	8.2
4		75.0	0.533	238.25	450.90	N/A	N/A	20.7	19.8
5		50.0	0.653	347.36	508.89	N/A	N/A	30.2	28.9
6		37.0	0.707	25.12	129.67	N/A	N/A	2.2	2.1
7		25.0	0.729	42.78	167.02	N/A	N/A	3.7	3.6
8		20.0	0.813	208.67	329.46	N/A	N/A	18.1	17.4
9		15.0	0.850	22.26	86.28	N/A	N/A	1.9	1.9
10		10.0	0.890	99.32	154.60	N/A	N/A	8.6	8.3

Band Detection	Automatically detected bands with sensitivity: High
Lane Background	Lane background subtracted with disk size: 0.1
Lane Width	5.02 mm
Regression Equation	y = -2.04 * x + 2.92<br>R-squared value: R^2 = 0.974032

Lane 2

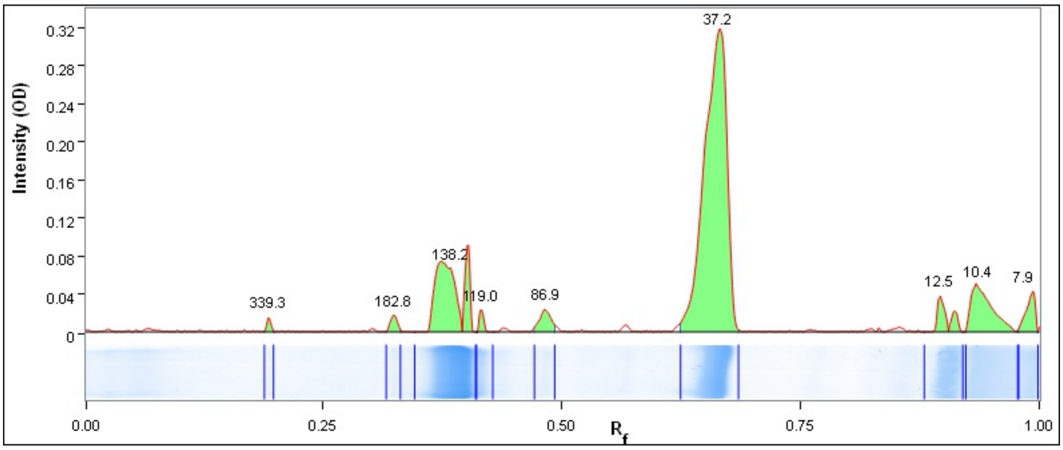

Band No.	Band Label	Mol. Wt. (KDa)	Relative Front	Adj. Volume (OD)	Volume (OD)	Abs. Quant.	Rel. Quant.	Band %	Lane %
1		339.3	0.193	6.39	17.01	N/A	N/A	0.5	0.5
2		182.8	0.324	13.03	44.46	N/A	N/A	1.1	1.0
3		138.2	0.384	203.69	1,440.15	N/A	N/A	17.0	16.3

4		119.0	0.416	10.88	147.32	N/A	N/A	0.9	0.9
5		86.9	0.483	27.84	58.07	N/A	N/A	2.3	2.2
6		37.2	0.664	748.00	823.64	N/A	N/A	62.3	59.8
7		12.5	0.897	42.82	407.99	N/A	N/A	3.6	3.4
8		10.4	0.936	107.90	478.20	N/A	N/A	9.0	8.6
9		7.9	0.994	39.73	167.38	N/A	N/A	3.3	3.2

Band Detection	Automatically detected bands with sensitivity: High
Lane Background	Lane background subtracted with disk size: 0.1
Lane Width	5.78 mm
Regression Equation	y = -2.04 * x + 2.92<br>R-squared value: R^2 = 0.974032

Lane 3

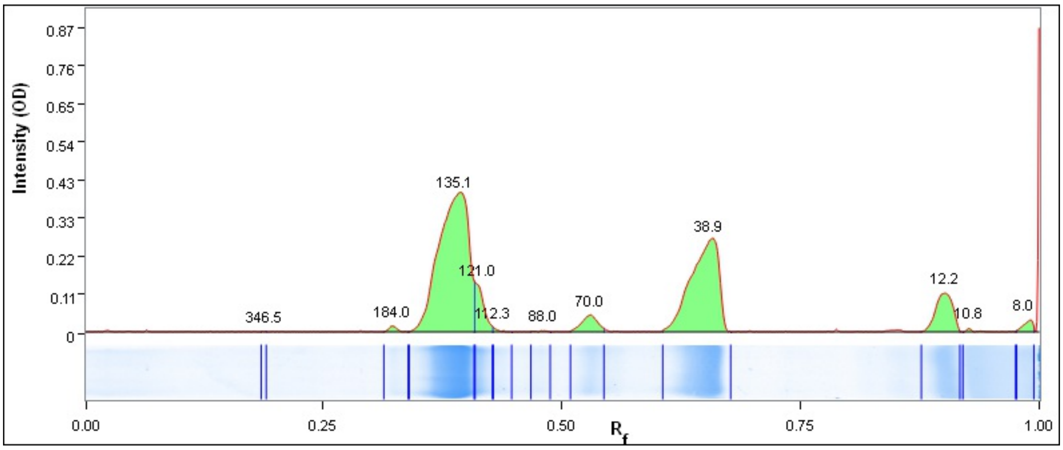

Band No.	Band Label	Mol. Wt. (KDa)	Relative Front	Adj. Volume (OD)	Volume (OD)	Abs. Quant.	Rel. Quant.	Band %	Lane %
1		346.5	0.188	0.46	12.11	N/A	N/A	0.0	0.0
2		184.0	0.323	13.54	82.20	N/A	N/A	0.5	0.5
3		135.1	0.389	1,261.35	1,502.04	N/A	N/A	50.3	47.7
4		121.0	0.412	129.28	204.13	N/A	N/A	5.2	4.9
5		112.3	0.428	5.39	78.41	N/A	N/A	0.2	0.2
6		88.0	0.480	2.76	65.00	N/A	N/A	0.1	0.1
7		70.0	0.529	79.79	176.97	N/A	N/A	3.2	3.0
8		38.9	0.655	777.06	918.24	N/A	N/A	31.0	29.4
9		12.2	0.901	200.85	404.46	N/A	N/A	8.0	7.6
10		10.8	0.927	6.42	477.86	N/A	N/A	0.3	0.2
11		8.0	0.990	33.20	151.73	N/A	N/A	1.3	1.3

Band Detection	Automatically detected bands with sensitivity: High
Lane Background	Lane background subtracted with disk size: 0.1
Lane Width	5.78 mm
Regression Equation	y = -2.04 * x + 2.92<br>R-squared value: R^2 = 0.974032

Lane 4

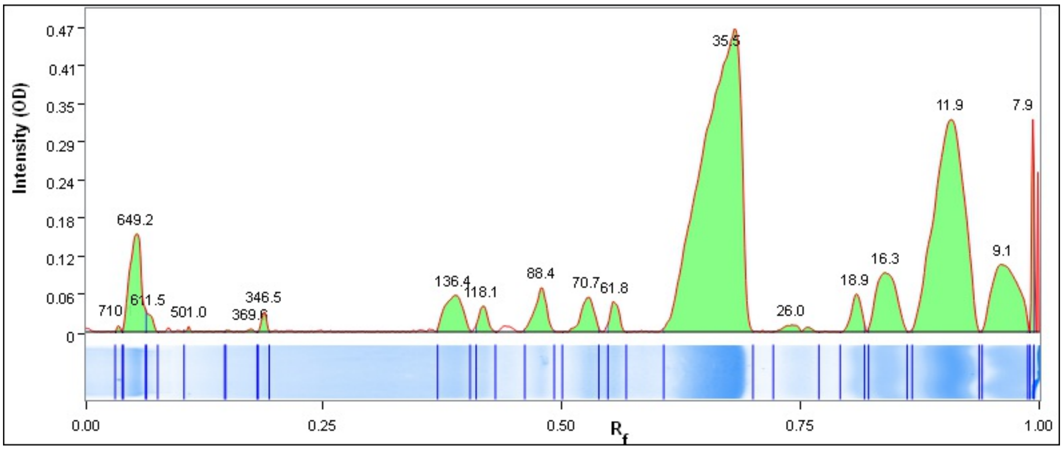

Band No.	Band Label	Mol. Wt. (KDa)	Relative Front	Adj. Volume (OD)	Volume (OD)	Abs. Quant.	Rel. Quant.	Band %	Lane %
1		710.1	0.035	3.23	51.21	N/A	N/A	0.1	0.1
2		649.2	0.054	187.50	303.20	N/A	N/A	4.5	4.4
3		611.5	0.067	18.75	59.34	N/A	N/A	0.5	0.4
4		501.0	0.109	4.36	197.35	N/A	N/A	0.1	0.1
5		369.6	0.174	3.83	224.21	N/A	N/A	0.1	0.1
6		346.5	0.188	16.20	107.77	N/A	N/A	0.4	0.4
7		136.4	0.387	107.73	403.58	N/A	N/A	2.6	2.5
8		118.1	0.418	36.65	162.39	N/A	N/A	0.9	0.9
9		88.4	0.479	83.85	178.28	N/A	N/A	2.0	2.0
10		70.7	0.527	72.30	313.95	N/A	N/A	1.7	1.7
11		61.8	0.556	42.75	191.25	N/A	N/A	1.0	1.0
12		35.5	0.674	1,882.32	2,189.35	N/A	N/A	45.3	44.5
13		26.0	0.741	21.75	212.07	N/A	N/A	0.5	0.5
14		18.9	0.809	60.76	215.43	N/A	N/A	1.5	1.4
15		16.3	0.840	198.00	567.95	N/A	N/A	4.8	4.7
16		11.9	0.908	1,047.37	1,534.09	N/A	N/A	25.2	24.8
17		9.1	0.964	291.57	699.93	N/A	N/A	7.0	6.9
18		7.9	0.994	79.07	156.33	N/A	N/A	1.9	1.9

Band Detection	Automatically detected bands with sensitivity: High
Lane Background	Lane background subtracted with disk size: 0.1
Lane Width	5.78 mm
Regression Equation	y = -2.04 * x + 2.92<br>R-squared value: R^2 = 0.974032

Lane 5

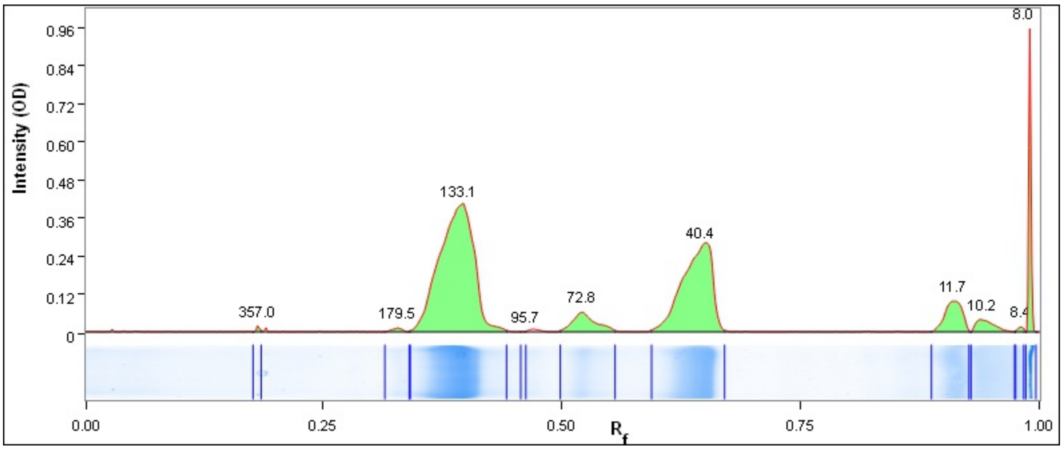

Band No.	Band Label	Mol. Wt. (KDa)	Relative Front	Adj. Volume (OD)	Volume (OD)	Abs. Quant.	Rel. Quant.	Band %	Lane %
1		357.0	0.182	6.17	18.38	N/A	N/A	0.2	0.2
2		179.5	0.328	12.74	79.58	N/A	N/A	0.4	0.4
3		133.1	0.392	1,462.73	1,756.24	N/A	N/A	48.9	48.3
4		95.7	0.462	0.48	17.48	N/A	N/A	0.0	0.0
5		72.8	0.521	146.05	236.54	N/A	N/A	4.9	4.8
6		40.4	0.646	872.40	968.22	N/A	N/A	29.2	28.8
7		11.7	0.911	180.97	359.11	N/A	N/A	6.0	6.0
8		10.2	0.940	72.80	370.40	N/A	N/A	2.4	2.4
9		8.4	0.981	8.93	61.57	N/A	N/A	0.3	0.3
10		8.0	0.990	228.01	255.80	N/A	N/A	7.6	7.5

Band Detection	Automatically detected bands with sensitivity: High
Lane Background	Lane background subtracted with disk size: 0.1
Lane Width	5.78 mm
Regression Equation	y = -2.04 * x + 2.92<br>R-squared value: R^2 = 0.974032

Lane 6

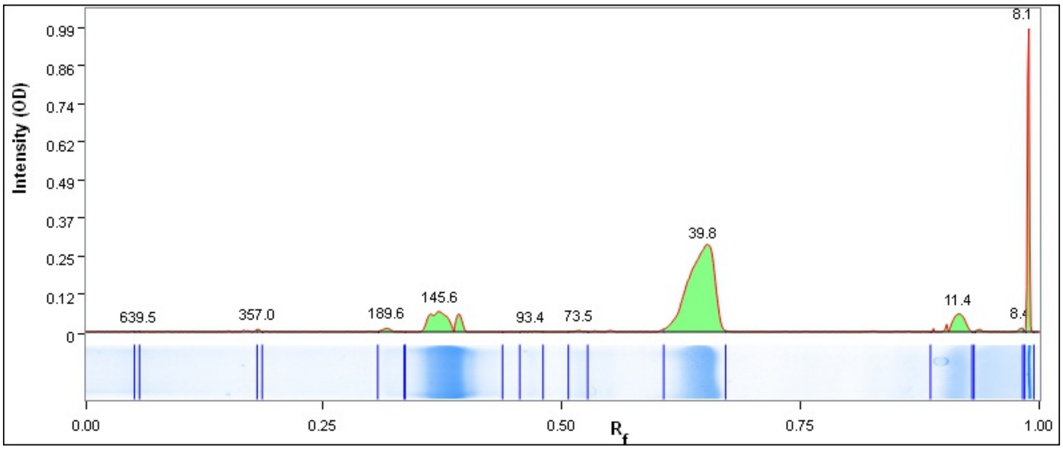

Band No.	Band Label	Mol. Wt. (KDa)	Relative Front	Adj. Volume (OD)	Volume (OD)	Abs. Quant.	Rel. Quant.	Band %	Lane %
1		639.5	0.057	0.49	21.79	N/A	N/A	0.0	0.0
2		357.0	0.182	2.00	8.86	N/A	N/A	0.2	0.2
3		189.6	0.317	10.30	74.91	N/A	N/A	0.8	0.8

4		145.6	0.373	164.45	1,644.85	N/A	N/A	13.1	12.8
5		93.4	0.468	2.09	54.38	N/A	N/A	0.2	0.2
6		73.5	0.519	2.96	61.38	N/A	N/A	0.2	0.2
7		39.8	0.649	776.54	851.74	N/A	N/A	61.9	60.2
8		11.4	0.916	78.48	358.58	N/A	N/A	6.3	6.1
9		8.4	0.982	9.93	367.01	N/A	N/A	0.8	0.8
10		8.1	0.989	207.31	229.70	N/A	N/A	16.5	16.1

Band Detection	Automatically detected bands with sensitivity: High
Lane Background	Lane background subtracted with disk size: 0.1
Lane Width	5.78 mm
Regression Equation	y = -2.04 * x + 2.92<br>R-squared value: R^2 = 0.974032

Lane 7

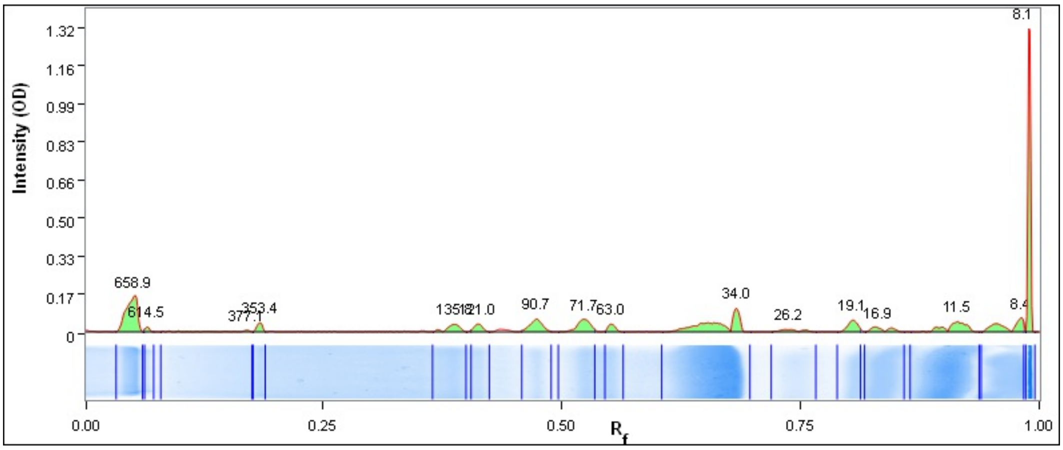

Band No.	Band Label	Mol. Wt. (KDa)	Relative Front	Adj. Volume (OD)	Volume (OD)	Abs. Quant.	Rel. Quant.	Band %	Lane %
1		658.9	0.051	187.75	350.85	N/A	N/A	13.8	13.4
2		614.5	0.066	9.50	51.31	N/A	N/A	0.7	0.7
3		377.1	0.170	10.53	590.22	N/A	N/A	0.8	0.8
4		353.4	0.184	23.41	129.61	N/A	N/A	1.7	1.7
5		135.8	0.388	42.83	386.13	N/A	N/A	3.1	3.1
6		121.0	0.412	30.60	149.02	N/A	N/A	2.2	2.2
7		90.7	0.474	72.33	162.92	N/A	N/A	5.3	5.2
8		71.7	0.524	74.61	296.57	N/A	N/A	5.5	5.3
9		63.0	0.552	25.98	174.54	N/A	N/A	1.9	1.9
10		34.0	0.683	204.08	2,189.35	N/A	N/A	15.0	14.6
11		26.2	0.739	20.96	199.45	N/A	N/A	1.5	1.5
12		19.1	0.806	52.06	185.30	N/A	N/A	3.8	3.7
13		16.9	0.832	35.23	540.98	N/A	N/A	2.6	2.5
14		11.5	0.915	91.39	1,446.72	N/A	N/A	6.7	6.5
15		8.4	0.981	98.49	592.76	N/A	N/A	7.2	7.0
16		8.1	0.989	380.24	418.78	N/A	N/A	28.0	27.2

Band Detection	Automatically detected bands with sensitivity: High
Lane Background	Lane background subtracted with disk size: 0.1
Lane Width	5.78 mm
Regression Equation	y = -2.04 * x + 2.92<br>R-squared value: R^2 = 0.974032

Lane 8

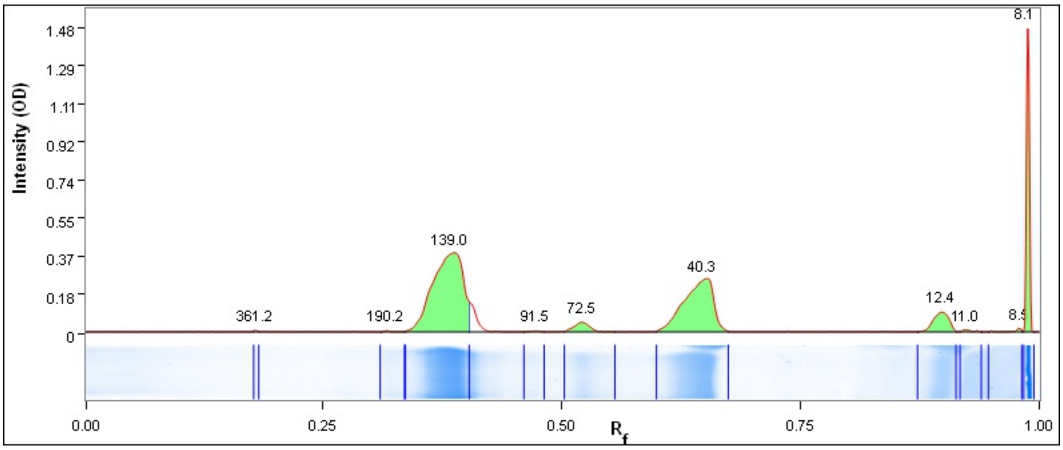

Band No.	Band Label	Mol. Wt. (KDa)	Relative Front	Adj. Volume (OD)	Volume (OD)	Abs. Quant.	Rel. Quant.	Band %	Lane %
1		361.2	0.179	2.28	6.07	N/A	N/A	0.1	0.1
2		190.2	0.316	3.23	78.64	N/A	N/A	0.1	0.1
3		139.0	0.383	1,245.13	1,477.75	N/A	N/A	44.7	42.7
4		91.5	0.472	4.07	56.84	N/A	N/A	0.1	0.1
5		72.5	0.522	76.14	218.45	N/A	N/A	2.7	2.6
6		40.3	0.647	797.51	909.12	N/A	N/A	28.6	27.4
7		12.4	0.898	160.10	394.14	N/A	N/A	5.7	5.5
8		11.0	0.924	9.36	214.67	N/A	N/A	0.3	0.3
9		8.5	0.980	9.59	249.26	N/A	N/A	0.3	0.3
10		8.1	0.988	480.12	501.52	N/A	N/A	17.2	16.5

Band Detection	Automatically detected bands with sensitivity: High
Lane Background	Lane background subtracted with disk size: 0.1
Lane Width	5.78 mm
Regression Equation	y = -2.04 * x + 2.92<br>R-squared value: R^2 = 0.974032

Lane 9

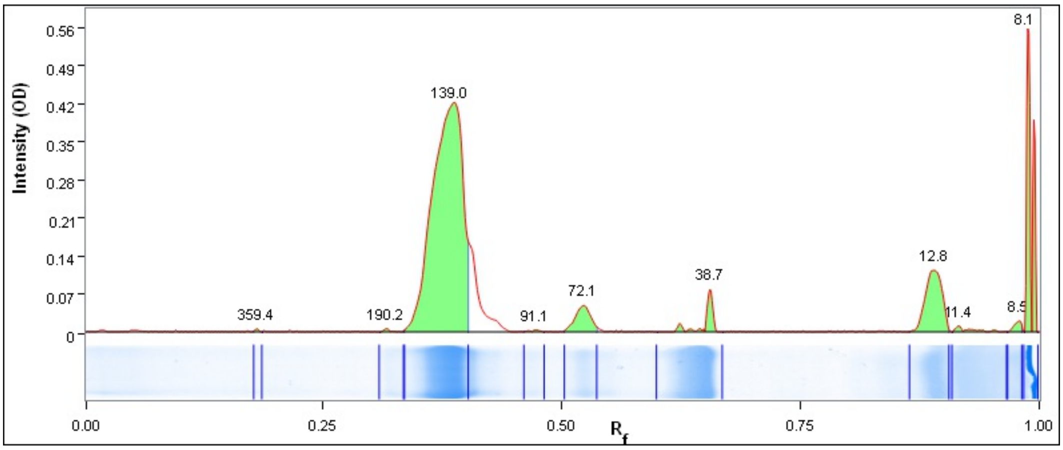

Band No.	Band Label	Mol. Wt. (KDa)	Relative Front	Adj. Volume (OD)	Volume (OD)	Abs. Quant.	Rel. Quant.	Band %	Lane %
1		359.4	0.180	2.09	11.52	N/A	N/A	0.1	0.1

2		190.2	0.316	3.61	75.05	N/A	N/A	0.2	0.2
3		139.0	0.383	1,357.16	1,532.36	N/A	N/A	66.6	60.5
4		91.1	0.473	3.14	55.11	N/A	N/A	0.2	0.1
5		72.1	0.523	79.34	155.92	N/A	N/A	3.9	3.5
6		38.7	0.655	57.69	904.55	N/A	N/A	2.8	2.6
7		12.8	0.891	204.38	410.46	N/A	N/A	10.0	9.1
8		11.4	0.916	14.27	539.17	N/A	N/A	0.7	0.6
9		8.5	0.979	15.20	129.36	N/A	N/A	0.7	0.7
10		8.1	0.989	299.95	648.98	N/A	N/A	14.7	13.4

Band Detection	Automatically detected bands with sensitivity: High
Lane Background	Lane background subtracted with disk size: 0.1
Lane Width	5.78 mm
Regression Equation	y = -2.04 * x + 2.92<br>R-squared value: R^2 = 0.974032
