# Supplementary material for: Larrea divaricata: anti-inflammatory and antioxidant effects of on macrophages and low density lipoproteins
Source: BMC Complement Med Ther. 2022 Mar 23;22:84. doi: 10.1186/s12906-022-03547-8 (PMC8941816; doi:10.1186/s12906-022-03547-8)
Supplement: Supplementary file 1 — Additional file 1. [file 12906_2022_3547_MOESM1_ESM.zip › Lipoprot purification gel 10.15.18.pdf]

Image Report: Lipoprot purification gel 10.15.18

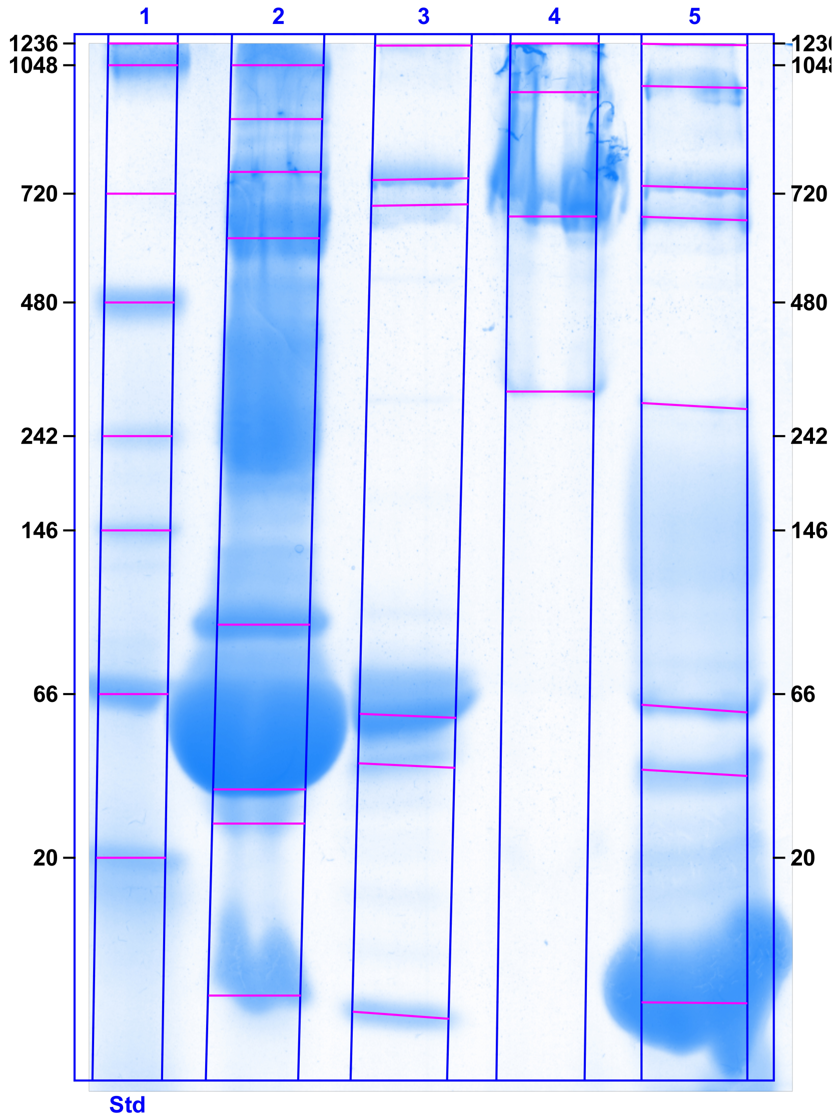

S:\Nako Peralta\Lipoprot purification gel 10.15.18.scn

Acquisition Information

Imager	GS-900™
Serial Number	SGCWBA10683
Firmware Version	61
Software Version	6.0.0.25
Application	Colloidal Coomassie
Scan Mode	Transmissive
Scan Color	Red
OD Calibration	Yes
Flat Field	Red

Image Information

Acquisition Date	10/15/2018 11:05:49 AM
User Name	Russell Caccavello
Image Area (mm)	X: 43.1 Y: 64.3
Pixel Size (µm)	X: 63.5 Y: 63.5
Data Range (OD)	0.000 - 1.599

Analysis Settings

Detection	Lane detection:<br>Manually created lanes<br><br>Band detection:<br>Automatically detected bands with sensitivity: Low<br>Manually adjusted bands<br><br>Lane Background Subtraction:<br>Lane background subtracted with disk size: 0.1<br><br>Lane width: Variable
Mol. Weight Analysis	Standard: NativeMark Invitrogen<br>Standard lanes: first<br>Regression method: Point to Point (semi-log)

Calibration Report

OD Value	Mean Intensity	Max Value	Min Value	Std Dev
0.00	6713	48712	4587	585.58
0.08	16737	34914	15040	600.76
0.27	32025	35501	30148	454.12
0.46	42848	45253	41345	339.60
0.69	50808	53313	49576	252.86
0.88	56025	57624	55272	176.71
1.13	59261	60007	58696	131.96
1.34	61332	61818	60915	97.53
1.55	62752	63231	62394	74.95
1.75	63603	63876	63348	59.27
1.97	64236	64436	63981	47.17
2.18	64629	64794	64468	39.72
2.39	64889	65018	64745	32.94
2.59	65044	65191	64891	28.37
2.83	65153	65274	65031	25.13
3.07	65228	65333	65099	23.03
3.30	65266	65389	65160	22.27
3.46	65279	65400	65165	23.22
3.64	65283	65405	65158	23.16
3.88	65290	65422	65185	24.14

Note: Values reported are specular OD values to account for differences in how scanner-based instruments measure diffuse and specular samples.

Lane Statistics

Lane No.	Adj. Total Band Vol. (OD)	Total Band Vol. (OD)	Adj. Total Lane Vol. (OD)	Total Lane Vol. (OD)	Bkgd. Vol. (OD)	Norm. Factor
1	1,683.03	3,273.73	1,629.62	6,164.74	4,535.12	N/A
2	1,885.80	17,011.15	1,834.85	29,651.57	27,816.73	N/A
3	2,388.97	4,164.26	2,499.07	5,473.32	2,974.25	N/A
4	356.03	3,362.02	208.59	5,437.35	5,228.76	N/A
5	2,549.43	9,243.50	2,673.81	14,515.70	11,841.88	N/A

Lane And Band Analysis

Lane 1 - NativeMark Invitrogen

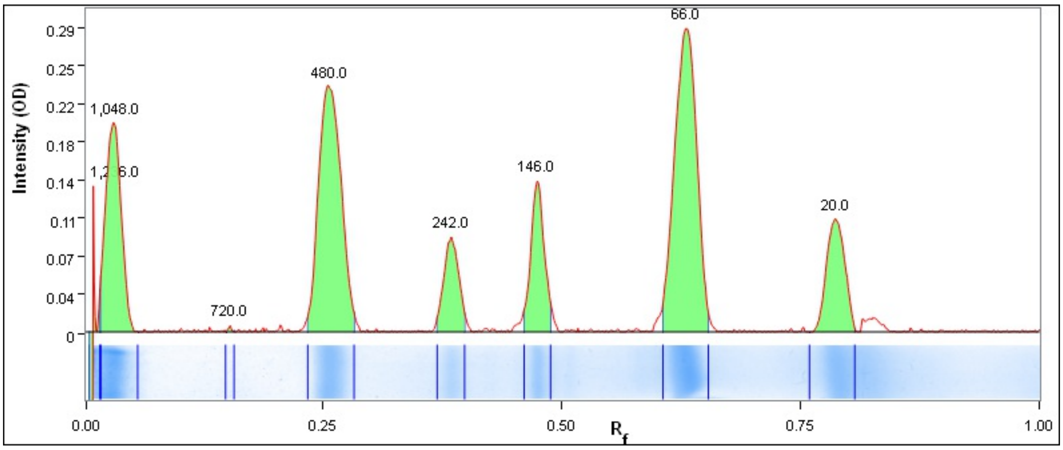

Band No.	Band Label	Mol. Wt. (KDa)	Relative Front	Adj. Volume (OD)	Volume (OD)	Abs. Quant.	Rel. Quant.	Band %	Lane %
1	IgM Hexamer	1,236.0	0.009	18.34	157.55	N/A	N/A	1.1	1.1
2	IgM Pentamer	1,048.0	0.030	255.75	651.36	N/A	N/A	15.2	15.7
3	Apoferritin band 1	720.0	0.152	1.49	17.91	N/A	N/A	0.1	0.1
4	Apoferritin band 2	480.0	0.256	439.82	533.25	N/A	N/A	26.1	27.0
5	B-phycoerythrin	242.0	0.384	111.24	219.21	N/A	N/A	6.6	6.8
6	Lactate Dehydrogenase	146.0	0.474	157.39	280.89	N/A	N/A	9.4	9.7
7	Bovine Serum Albumin	66.0	0.631	534.24	795.74	N/A	N/A	31.7	32.8
8	Soybean Trypsin Inhibitor	20.0	0.787	164.76	617.81	N/A	N/A	9.8	10.1

Band Detection	Automatically detected bands with sensitivity: Low
Lane Background	Lane background subtracted with disk size: 0.1
Lane Width	4.25 mm
Regression Equation	A single equation is not available for this method

Lane 2

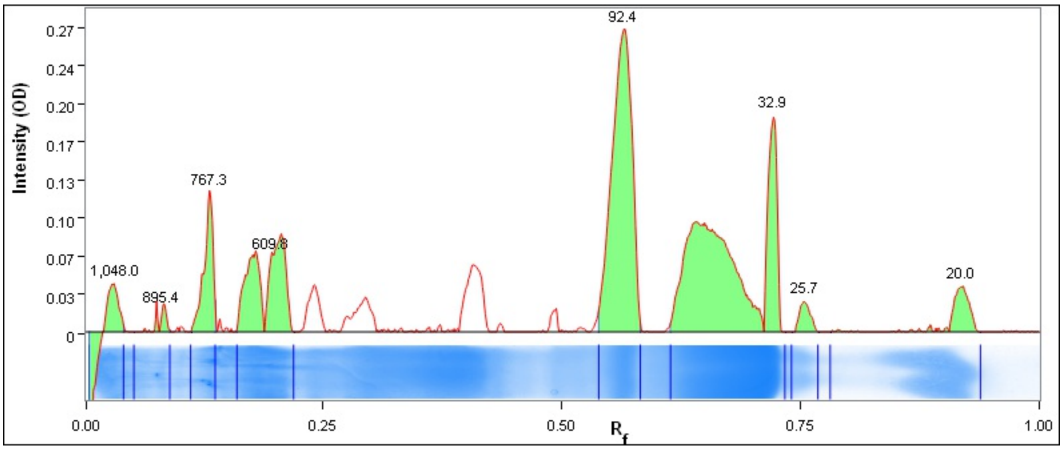

Band No.	Band Label	Mol. Wt. (KDa)	Relative Front	Adj. Volume (OD)	Volume (OD)	Abs. Quant.	Rel. Quant.	Band %	Lane %
1		1,048.0	0.030	51.47	1,102.39	N/A	N/A	2.7	2.8
2		895.4	0.081	19.05	813.99	N/A	N/A	1.0	1.0
3		767.3	0.132	120.87	760.04	N/A	N/A	6.4	6.6
4		609.8	0.195	264.79	2,545.87	N/A	N/A	14.0	14.4
5		92.4	0.564	593.39	1,567.00	N/A	N/A	31.5	32.3
6		32.9	0.722	729.86	7,564.06	N/A	N/A	38.7	39.8
7		25.7	0.754	32.31	438.38	N/A	N/A	1.7	1.8
8		20.0	0.919	74.06	2,219.43	N/A	N/A	3.9	4.0

Band Detection	Automatically detected bands with sensitivity: Low
Lane Background	Lane background subtracted with disk size: 0.1
Lane Width	5.65 mm
Regression Equation	A single equation is not available for this method

Lane 3

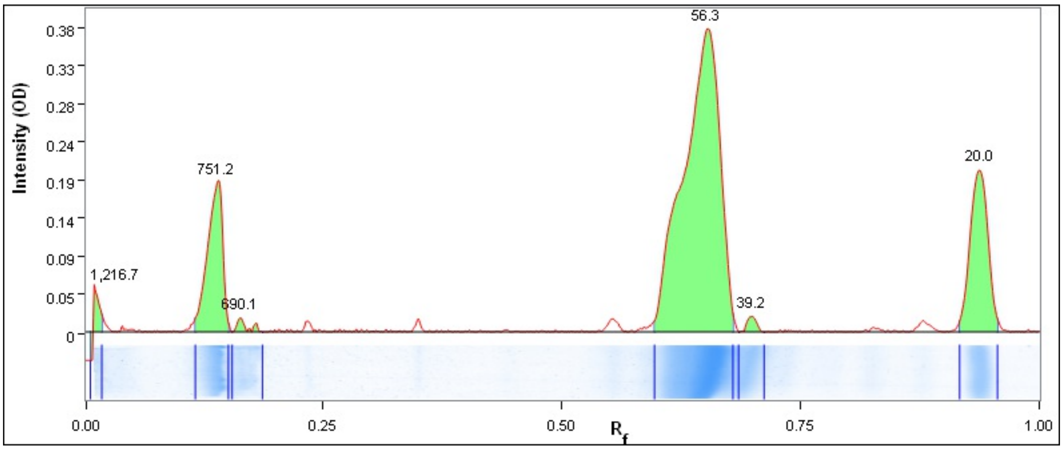

Band No.	Band Label	Mol. Wt. (KDa)	Relative Front	Adj. Volume (OD)	Volume (OD)	Abs. Quant.	Rel. Quant.	Band %	Lane %
1		1,216.7	0.011	36.87	73.30	N/A	N/A	1.5	1.5
2		751.2	0.139	327.55	514.42	N/A	N/A	13.7	13.1
3		690.1	0.163	18.91	241.86	N/A	N/A	0.8	0.8
4		56.3	0.651	1,567.25	2,495.50	N/A	N/A	65.6	62.7
5		39.2	0.699	20.84	401.00	N/A	N/A	0.9	0.8
6		20.0	0.938	417.55	438.19	N/A	N/A	17.5	16.7

Band Detection	Automatically detected bands with sensitivity: Low
Lane Background	Lane background subtracted with disk size: 0.1
Lane Width	5.91 mm
Regression Equation	A single equation is not available for this method

Lane 4

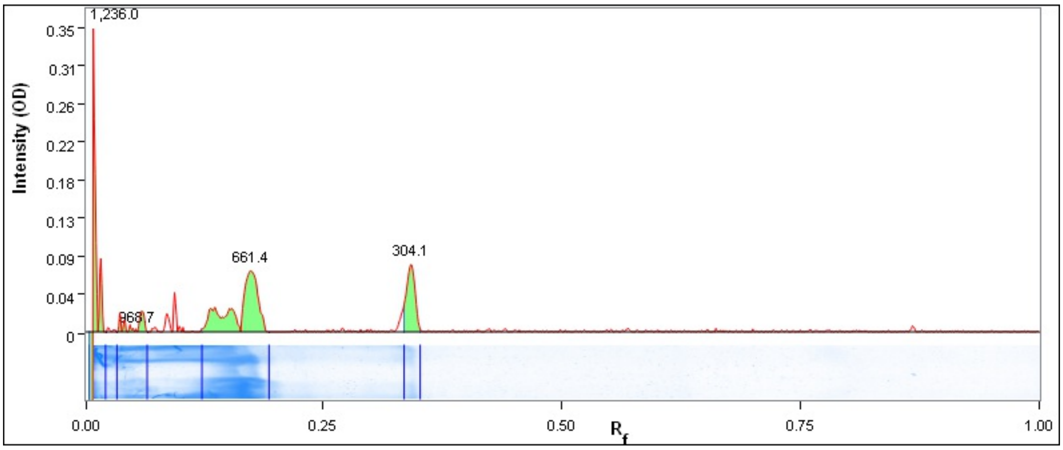

Band No.	Band Label	Mol. Wt. (KDa)	Relative Front	Adj. Volume (OD)	Volume (OD)	Abs. Quant.	Rel. Quant.	Band %	Lane %
1		1,236.0	0.009	100.40	470.24	N/A	N/A	28.2	48.1
2		968.7	0.055	21.16	627.94	N/A	N/A	5.9	10.1
3		661.4	0.174	163.27	2,148.64	N/A	N/A	45.9	78.3
4		304.1	0.342	71.19	115.20	N/A	N/A	20.0	34.1

Band Detection	Automatically detected bands with sensitivity: Low
Lane Background	Lane background subtracted with disk size: 0.1
Lane Width	5.40 mm
Regression Equation	A single equation is not available for this method

Lane 5

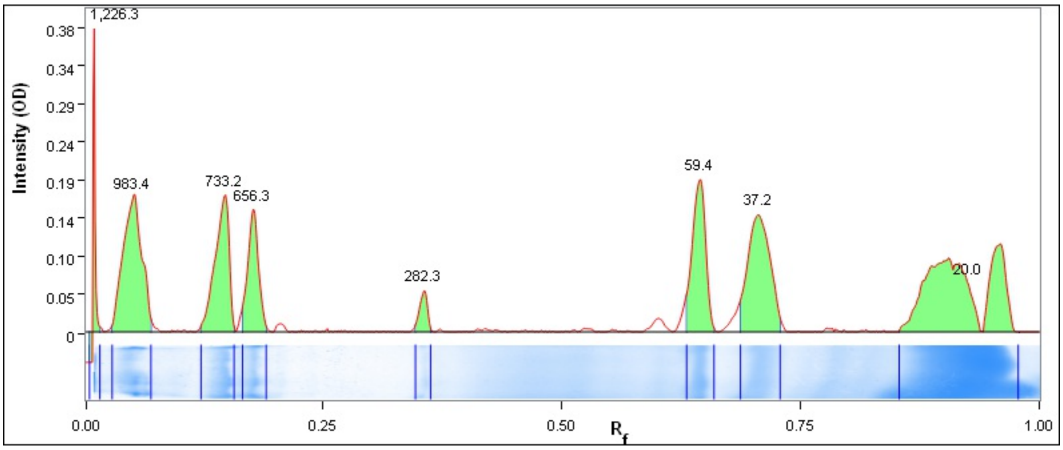

Band No.	Band Label	Mol. Wt. (KDa)	Relative Front	Adj. Volume (OD)	Volume (OD)	Abs. Quant.	Rel. Quant.	Band %	Lane %
1		1,226.3	0.010	94.59	126.12	N/A	N/A	3.7	3.5
2		983.4	0.050	409.47	580.94	N/A	N/A	16.1	15.3
3		733.2	0.147	304.42	539.57	N/A	N/A	11.9	11.4
4		656.3	0.176	211.38	380.37	N/A	N/A	8.3	7.9
5		282.3	0.355	52.09	110.40	N/A	N/A	2.0	1.9
6		59.4	0.645	325.90	514.00	N/A	N/A	12.8	12.2
7		37.2	0.706	419.07	661.34	N/A	N/A	16.4	15.7
8		20.0	0.926	732.53	6,330.75	N/A	N/A	28.7	27.4

Band Detection	Automatically detected bands with sensitivity: Low

Lane Background	Lane background subtracted with disk size: 0.1
Lane Width	6.48 mm
Regression Equation	A single equation is not available for this method
