# Supplementary figures and images for: Larrea divaricata: anti-inflammatory and antioxidant effects of on macrophages and low density lipoproteins
Source: BMC Complement Med Ther. 2022 Mar 23;22:84. doi: 10.1186/s12906-022-03547-8 (PMC8941816; doi:10.1186/s12906-022-03547-8)

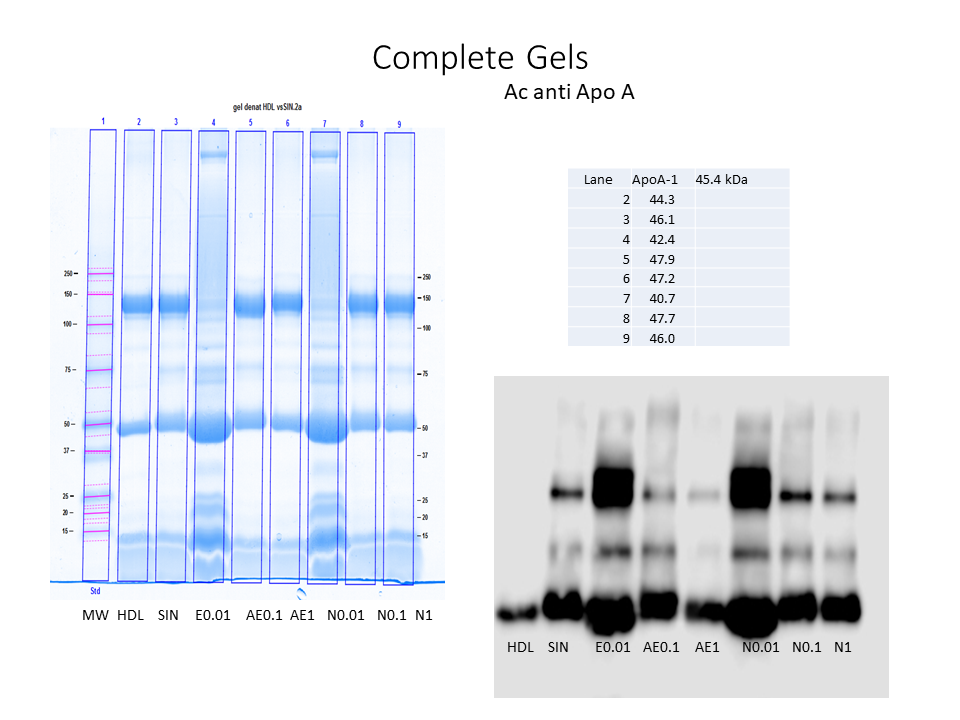

Supplement: Supplementary file 1 — Additional file 1. [file 12906_2022_3547_MOESM1_ESM.zip › complet gels.tif]
